# Crosstalk between TGF-β and Wnt/β-catenin signaling drives fibrogenic and stem-like phenotypes in senescent MDA-MB-231 breast cancer cells

**DOI:** 10.1038/s41514-025-00322-0

**Published:** 2026-01-03

**Authors:** Mona El Samarji, Elissa Alam, Mariam Dakramanji, Mariam Bassam, Jana Santina, Marc Ayoub, Alex Aprahamian, Mohamad Rima

**Affiliations:** Department of Biological Sciences, Lebanese American University, Byblos, Lebanon

**Keywords:** Cancer, Cell biology, Molecular biology, Stem cells

## Abstract

Genotoxic drugs used to treat cancer can trigger senescence, which contributes to chemotherapy resistance and tumor heterogeneity. However, the resulting cellular and molecular alterations following senescence remain poorly characterized. In this study, chemotherapy-induced senescence was triggered by etoposide in MDA-MB-231 breast cancer cells, and their fibrogenic potential, epithelial-to-mesenchymal transition (EMT), and stemness features were examined. In these cells, key mediators of fibrosis were significantly upregulated, suggesting a profibrotic potential involving TGF-β signaling. Etoposide also accentuated the mesenchymal phenotype of MDA-MB-231 cells and increased their motility. Additionally, nuclear β-catenin accumulation and upregulation of its EMT target genes were observed in senescent cells, alongside increased stemness markers, indicating a plastic cellular state involving Wnt/β-catenin signaling. Interestingly, pharmacological inhibition of the TGF-β/Wnt/β-catenin pathways reduced fibrosis, EMT, stemness marker expression, and cell migration, suggesting that these pathways are key regulators of these processes in senescent cells. These findings provide new insights into the molecular mechanisms driving chemotherapy-induced senescence and highlight these pathways as potential targets to alleviate resistance and aggressiveness in breast cancer.

## Introduction

Cellular senescence is a state of stable cell cycle arrest triggered by various intrinsic and extrinsic factors. This state is characterized by several hallmarks including (i) enlarged cell morphology with increased cytoplasmic granularity, (ii) induction of senescence-associated beta-galactosidase (SA-β-gal) activity^1^, (iii) aberrant metabolic activity, (iv) absence of proliferation^2^, and (v) secretion of the senescence-associated secretory phenotype (SASP) factors, which include interleukins, chemokines, and proteases^3^. The SASP has paradoxical roles, mediating processes like wound healing^4,5^, developmental senescence^6,7^, chronic inflammation^8^, cellular plasticity^9^, and aging^10^. While replicative senescence naturally occurs due to telomere shortening aiming to limit the reproductive lifespan of normal cells^11,12^, stress-induced senescence (also known as accelerated or premature senescence) has been described as a response to different stressors including telomere erosion, DNA damage, protein misfolding, oncogene activation, oxidative stress, extracellular signals from mitogens or cytokines, and a high-fat diet^2,13^. For example, upon oncogene activation, senescence acts as a tumor-suppressive mechanism by halting cell proliferation and preventing malignant progression^14^. Consequently, senescence has been described as a key strategy in many anticancer therapies, particularly chemotherapeutic agents that induce genotoxic stress to activate the senescence program^15–17^. Conversely, therapy-induced senescence can also result in chronic inflammation, drug resistance^18^, and pro-aging effects^10^, which in turn increase the risk of cancer development^19,20^ and age-related diseases^21^. Although senescence serves as a barrier to tumorigenesis, it has been suggested that senescent cells escaping immune system elimination and remaining dormant in vivo may eventually reenter the cell cycle, leading to tumor regrowth and disease recurrence^22^. This dual role arises primarily due to the SASP that recruits immune cells to eliminate senescent cells. However, in cases where senescent cells are not effectively cleared, the SASP can create a pro-inflammatory microenvironment that supports tumor growth, enhances angiogenesis, and promotes cancer cell plasticity through the acquisition of stem-like properties^23^.

Several chemotherapeutic drugs (etoposide, cisplatin, doxorubicin…) used in cancer therapy can elicit two distinct cellular outcomes: an apoptotic programmed cell death in response to severe DNA damage, or a senescent state of cell cycle arrest following sublethal genotoxic stress^19,24–26^. For example, etoposide, a topoisomerase II inhibitor, is widely used for malignancy chemotherapy that couples DNA damage to cellular apoptosis^27^. At sub-cytotoxic doses, this drug leads to senescence by triggering DNA damage response pathway^28–31^. Numerous in vitro, in vivo, and clinical studies investigated senescence in breast cancer treatments^32^. Among these studies, etoposide was shown to induce senescence in breast cancer cell models: MCF-7, a cell line model of luminal breast cancer, and MDA-MB-231, a model of basal-like carcinoma with aggressive, invasive, and chemoresistant properties^16,33–35^. Despite widespread use of etoposide to induce senescence in MDA-MB-231 breast cancer cells, the resulting cellular and molecular alterations remain poorly characterized. While some individual pathways are known to be upregulated in these senescent cells, the potential crosstalk between signaling pathways and their roles in driving stemness-like properties and EMT in senescent breast cancer cells has not been explored yet. This study aims to establish a robust model of etoposide-induced senescence in MDA-MB-231 cells and to explore the associated phenotypic and molecular changes. After validating the cellular model of senescence, we investigated its profibrotic potential, especially given that the aberrant and chronic accumulation of senescent cells drives various features of aging, including age-related fibrosis^36^. In addition, since MDA-MB-231 cells are well known for their mesenchymal properties and are frequently used to study EMT in cancer research^37^, we also investigated the potential of etoposide to affect the mesenchymal status in these cells and characterized the corresponding signaling pathways activated in response to the treatment. Our study enhances the understanding of chemotherapy-induced senescence and offers valuable insights for improving cancer treatment management strategies.

## Results

### Validation of chemotherapy-induced senescence in MDA-MB-231 breast cancer cells

**Fig. 1:**
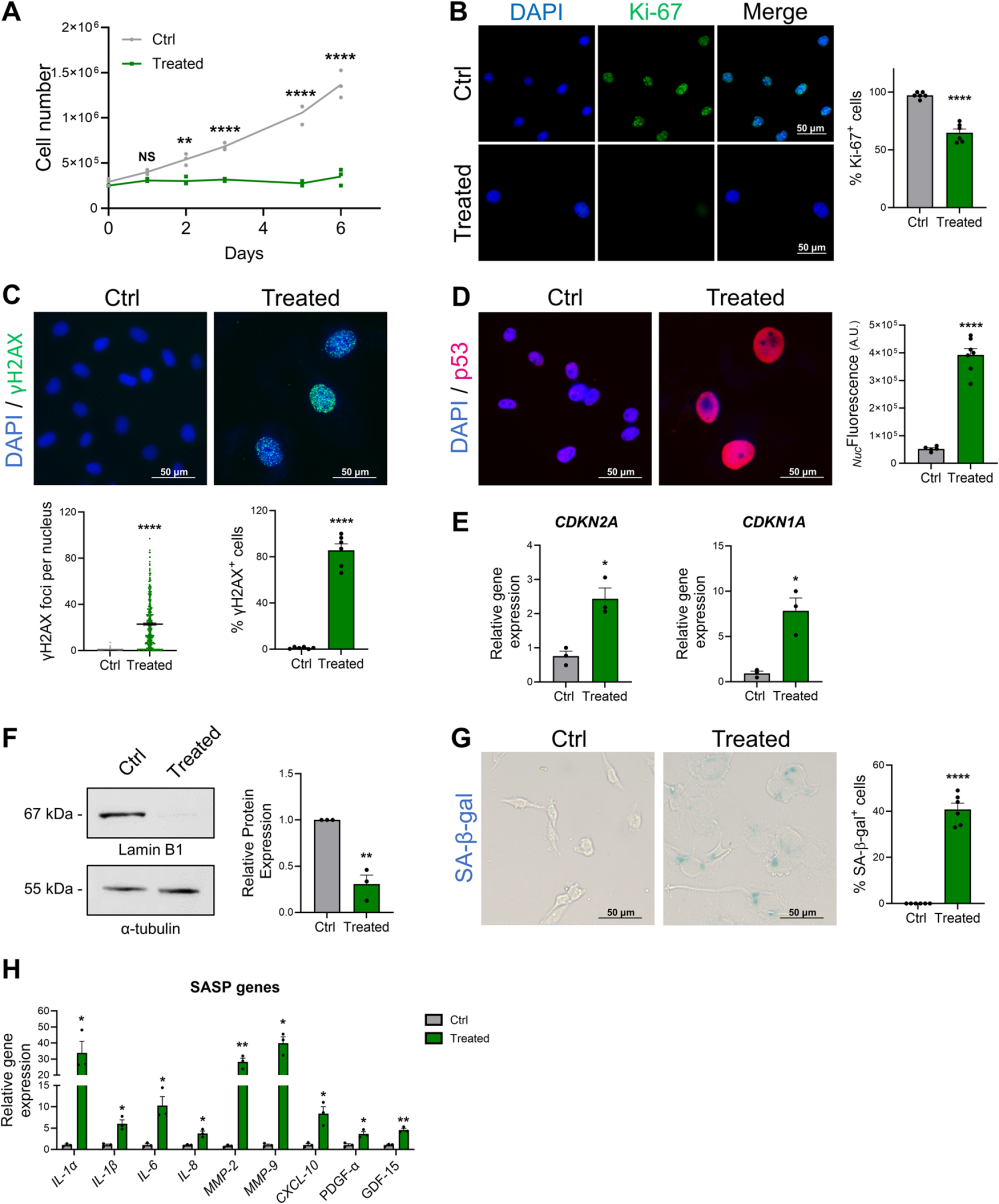
Development and validation of etoposide-induced MDA-MB-231 senescence model. **A** Cell growth curve showing cell count variation in both control and etoposide-treated MDA-MB-231 conditions. **B** Representative images and quantification of Ki-67 expression (green) in MDA-MB-231 cells six days after etoposide treatment. The nucleus was counterstained with DAPI. The graph shows the percentage of cycling (Ki-67^+^) cells. **C** Representative images and quantification of γH2AX foci (green) in control and etoposide-treated MDA-MB-231 cells. The nucleus was counterstained with DAPI. The graphs represent the number of foci per nucleus (left), and the percentage of γH2AX^+^ cells (right), corresponding to the cells showing more than 1 focus. **D** Representative images and quantification of p53 (red) in MDA-MB-231 cells after etoposide treatment. The nucleus was counterstained with DAPI. The graph represents the nuclear fluorescence intensity of p53 staining. **E** qRT-PCR analysis of p16 (*CDKN2A*) and p21 (*CDKN1A*) mRNA expression levels after etoposide treatment. **F** Western blot analysis of Lamin B1 expression in MDA-MB-231 after etoposide treatment. α-tubulin blot was used as loading control. The original blots are presented in Supplementary Figure 2. **G** Representative images and quantification of SA-β-gal staining in control and etoposide-treated MDA-MB-231 cells. **H** qRT-PCR analysis of SASP: *IL-1α, IL-1β, IL-6, IL-8, CXCL-10, MMP-2, MMP-9, GDF-15*, *PDGF-α* mRNA expression levels after etoposide treatment. NS: not significant; *p ≤ 0.05;**p ≤ 0.01;****p ≤ *0.0001*.

To validate the induction of senescence in MDA-MB-231 cells after etoposide treatment, proliferation, DNA damage, and senescence markers expression were evaluated. The cell counting assay revealed that the number of etoposide-treated cells remained relatively constant over the 6-days period post-treatment, in contrast to untreated cells, which exhibited a normal exponential proliferation rate (Fig. 1A), indicating that etoposide significantly inhibited cell proliferation. To validate this finding, the proliferation marker Ki-67 was assessed by immunofluorescence. In etoposide-treated condition, the percentage of Ki-67^+^ cells significantly decreased, compared to the control group (Fig. 1B). Of note, at this concentration of etoposide (2.5 µM), no signs of cytotoxicity were detected, as indicated by the very low percentage (<5%) of Annexin V/PI-positive cells (Supplementary Fig. 1).

Since etoposide is known to induce DNA damage by interacting with the nuclear enzyme topoisomerase II^38^, we examined the appearance of γH2AX foci as an indicator for DNA damage. Immunofluorescence images show a significant increase in the percentage of γH2AX^+^ cells and γH2AX foci number per cell upon treatment (Fig. 1C). Etoposide-treated cells displayed also higher nuclear p53 expression, compared to control cells (Fig. 1D), and significant upregulation of p16 (*CDKN2A)* and p21 (*CDKN1A)* transcripts (Fig. 1E), suggesting cell cycle arrest that is normally triggered by the activation of DNA Damage Response (DDR). Together, these findings suggest that at non-cytotoxic concentration of etoposide, the drug induces DNA damage, halts the cell cycle and blocks cell proliferation, all of which are hallmarks of cellular senescence.

Next, we further investigated the senescence phenotype of MDA-MB-231 breast cancer cell line by checking the expression of several senescence markers. First, western blot analysis shows a significant downregulation of Lamin B1, the nuclear envelope integrity marker, in treated cells (Fig. 1F). Furthermore, SA-β-gal staining revealed a significant increase in the percentage of SA-β-gal⁺ cells in the etoposide-treated group compared to the control (Fig. 1G). We also assessed SASP expression by qRT-PCR which shows a significant upregulation of interleukins (*IL-1α, IL-1β, IL-6, IL-8*), chemokines (*CXCL-10*), and matrix metalloproteinases (*MMP-2* and *MMP-9*) mRNA in etoposide-treated cells (Fig. 1H), suggesting the full establishment of the SASP. Other SASP markers such as GDF-15 (Growth and Differentiation Factor 15) and PDGF-α (Platelet-Derived Growth Factor) were also significantly upregulated in etoposide-treated cells (Fig. 1H). Collectively, these findings highlight the senescence phenotype of MDA-MB-231 cells induced by non-cytotoxic concentrations of etoposide, thereby validating the establishment of genotoxic stress-induced premature senescence of MDA-MB-231 human breast cancer cells. Accordingly, etoposide-treated MDA-MB-231 cells are referred to as senescent (Sen), while control counterparts are termed proliferating (Pro) in the following sections.

### Profibrotic potential of etoposide-induced senescent MDA-MB-231 cells

**Fig. 2:**
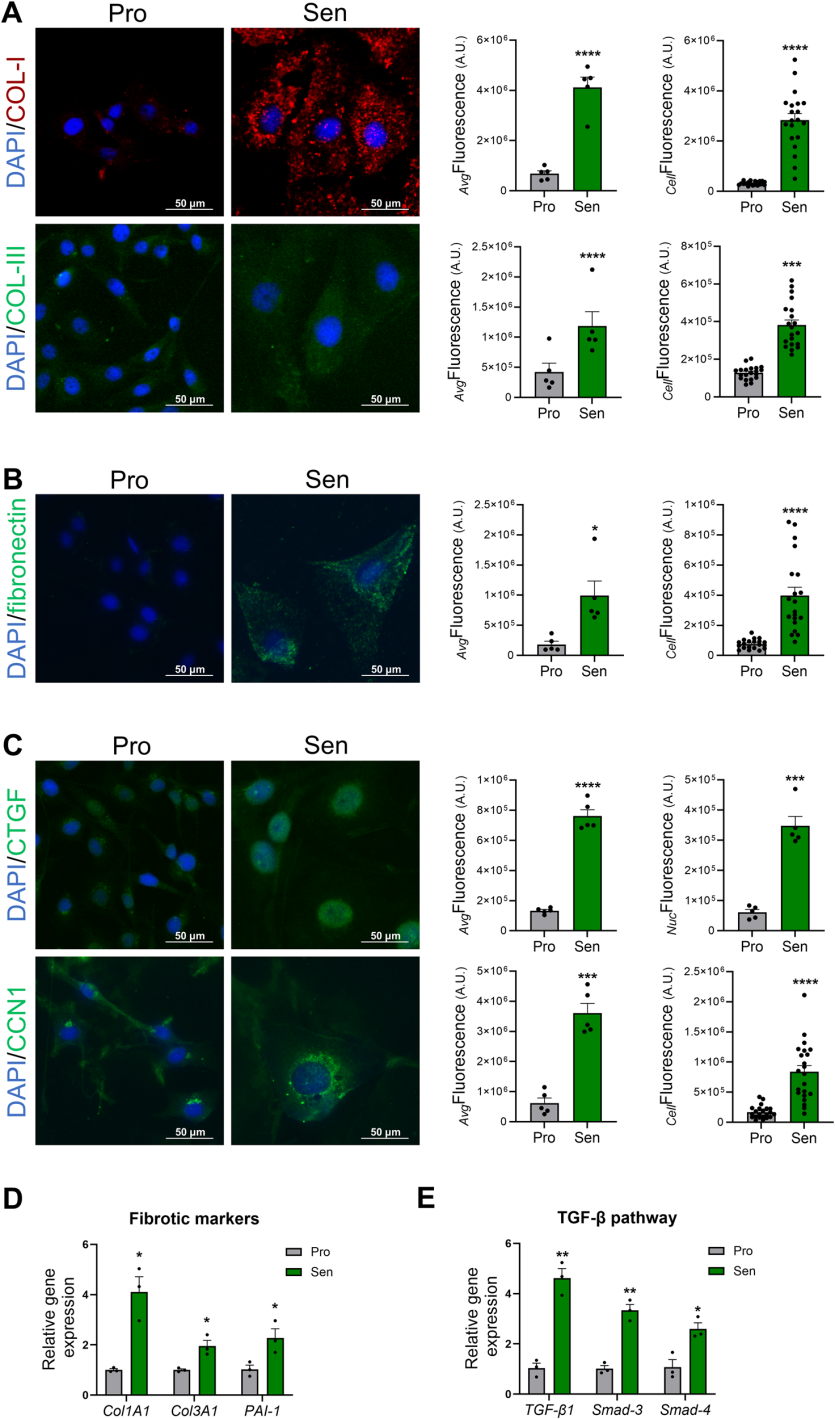
Upregulation of central mediators of tissue remodeling and fibrotic markers in etoposide-induced MDA-MB-231 senescence model. **A** Representative images and quantification of collagen type 1 (COL-1, red) and type 3 (COL-3, green) in MDA-MB-231 cells six days after etoposide treatment. The nucleus was counterstained with DAPI. The graphs represent the average fluorescence intensity per cell (_*Avg*_Fluorescence) and cellular fluorescence (_*Cell*_Fluorescence), two different quantification methods, described in the “Methods” section, that yield similar results. **B** Representative images and quantification of fibronectin (green) in eto-induced MDA-MB-231 senescent cells. The nucleus was counterstained with DAPI. The graphs represent the average fluorescence intensity per cell (_*Avg*_Fluorescence) and cellular fluorescence (_*Cell*_Fluorescence). **C** Representative images and quantification of the Connective Tissue Growth Factor (CTGF) and the Cellular Communication Network factor 1 (CCN1) (green) in control (proliferating) and etoposide-induced MDA-MB-231 senescent cells. The nucleus was counterstained with DAPI. The graphs represent the average fluorescence intensity per cell (_*Avg*_Fluorescence) for the two proteins along with the nuclear fluorescence (_*Nuc*_Fluorescence) for CTGF, and cellular fluorescence (_*Cell*_Fluorescence) for CCN1. Of note, _*Avg*_Fluorescence and _*Cell*_Fluorescence yield similar results. **D**, **E** qRT-PCR analysis showing changes in the mRNA levels of fibrotic markers (**D**) and TGF-β signaling pathway components (**E**) in etoposide-induced senescent MDA-MB-231 cells. *p ≤ 0.05;**p ≤ 0.01;***p ≤ 0.001;****p ≤ *0.0001*.

Since senescence is associated with organ-specific fibrosis^39^, we investigated whether etoposide treatment alters ECM composition and may therefore exert fibrotic effects. This hypothesis stems from the fact that, within the upregulated SASP markers of this senescence model, the central mediators of tissue remodeling, GDF-15 and PDGF-α (Fig. 1H), are known as central contributors to fibrosis in several types of tissue^40–43^. Immunofluorescence images, their quantifications, and qRT-PCR results show that collagen-I and collagen-III levels were significantly higher in senescent MDA-MB-231 cells (Fig. 2A, D), suggesting an increased production of extracellular matrix components, a master feature of tissue fibrosis. Of note, the two methods used to quantify immunofluorescence images, as described in the “Methods” section, yielded comparable results; however, some subsequent quantifications were randomly performed using either method.

To further confirm the profibrotic response following senescence induction, mRNA level of Plasminogen Activator Inhibitor-Type1 (PAI-1), which is implicated in the pathology of fibrosis, was assessed and found to be significantly upregulated in senescent cells (Fig. 2D). Fibronectin expression was further examined by immunofluorescence and was found to be increased in senescent MDA-MB-231 cells (Fig. 2B). To verify that this increase reflects elevated fibronectin levels within the extracellular matrix (ECM), immunofluorescence staining was also performed on decellularized coverslips. This approach allowed for visualization and quantification of fibronectin present in the ECM independent of cellular components. The immunofluorescence images demonstrated an increase in fibronectin levels in decellularized senescent conditions compared to control proliferating cells (Supplementary Fig. 3). These results suggest that the upregulation of fibronectin synthesis in senescent cells leads to enhanced fibronectin accumulation in the ECM, potentially impacting the cellular microenvironment. Together, these findings suggest that senescence accompanying etoposide treatment induces ECM thickening with increased collagen and fibronectin deposition, hallmarks of fibrosis.

Growing evidence links GDF-15 to senescence-associated fibrosis and ECM remodeling^43,44^. GDF-15 is a distant member of the Transforming Growth Factor-beta (TGF-β) superfamily^45^, sharing structural and signaling features with canonical TGF-β ligands^46^. Therefore, we analyzed the expression of several mediators of the fibrotic response of TGF-β signaling^47,48^, including Cellular Communication Network factor 1 (CCN1), Connective Tissue Growth Factor (CTGF), *Smad-3*, and *Smad-4*^49,50^. Immunofluorescence images showed a significant increase in CCN1 and CTGF levels in senescent cells, which was further confirmed by quantifications of fluorescence intensity (Fig. 2C). Interestingly, the upregulation of CTGF levels was not only observed throughout the cell but was also detected in the nucleus of senescent cells, which aligns with the non-canonical functions of CTGF. The increase of CCN1 and CTGF levels suggests an activation of TGF-β signaling, which was further confirmed by qRT-PCR showing a significant upregulation of key elements of the pathway: *TGF-β1*, *Smad-3*, and *Smad-4* in the fibrotic response (Fig. 2E), as it is a critical pathway for generating fibrotic tumor microenvironment (TME)^49^. Together, these findings show that etoposide-induced senescence in MDA-MB-231 cells is accompanied with the upregulation of ECM components synthesis and increased TGF-β signaling, both markers of tissue fibrosis.

### Etoposide-induced senescence accentuates the mesenchymal phenotype of MDA-MB-231 cells

In cancer, TGF-β plays a critical role in TME by accelerating invasion and metastasis^49^. In addition, MDA-MB-231 are classified as mesenchymal-like cancer cells with enhanced motility and invasiveness. Therefore, we investigated whether their commitment to senescence would affect their mesenchymal-like and motility profiles. To this end, the expression of several epithelial and mesenchymal markers was assessed by immunofluorescence, western blot, and qRT-PCR.

**Fig. 3:**
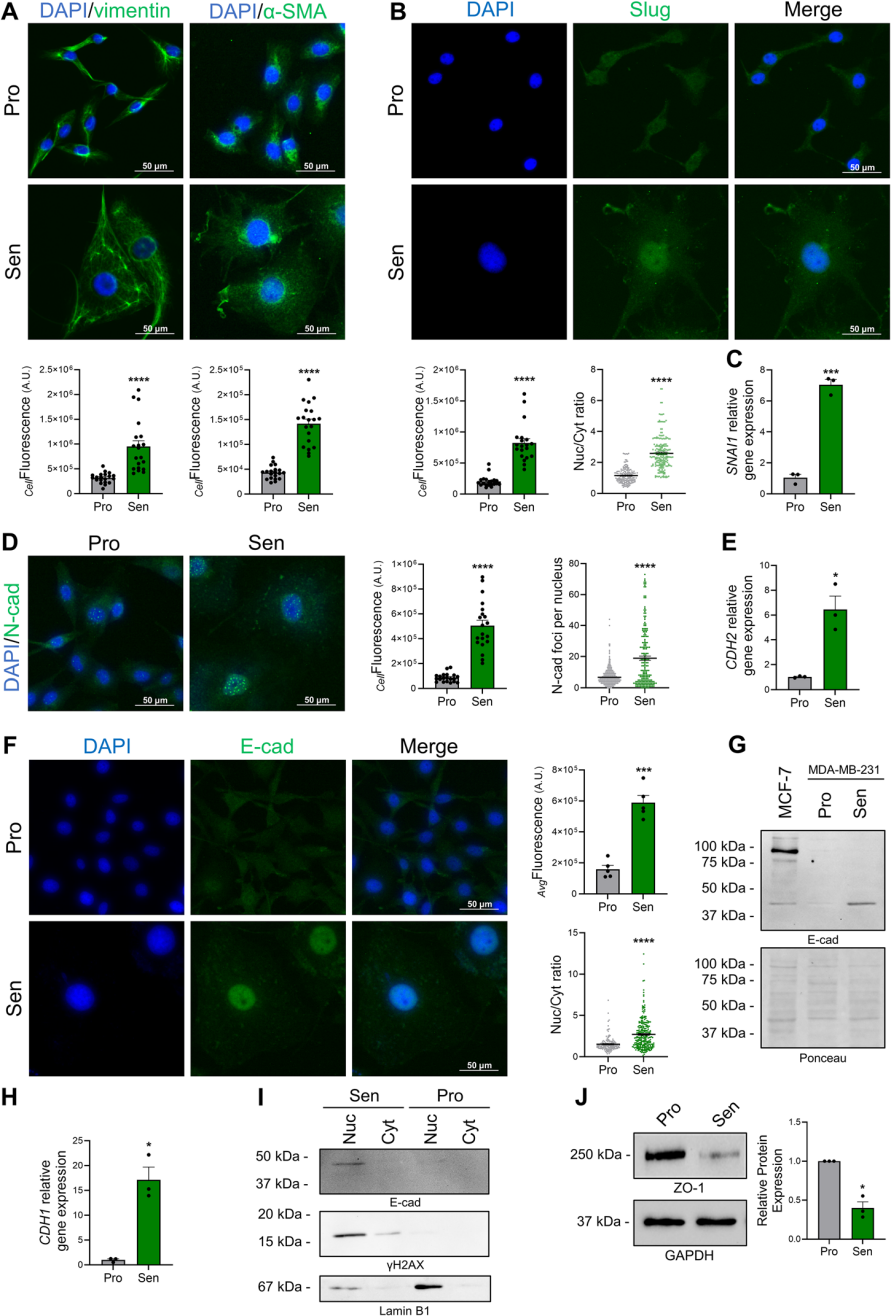
Etoposide treatment enhances the mesenchymal phenotype of MDA-MB-231 cells. **A** Representative images and quantification of vimentin and α-smooth muscle actin (α-SMA) (green) in MDA-MB-231 cells six days after etoposide treatment. The nucleus was counterstained with DAPI. The graphs represent the cellular fluorescence (_*Cell*_Fluorescence). **B** Representative images and quantification of the transcription factor Slug (green) in MDA-MB-231 cells after etoposide treatment. The nucleus was counterstained with DAPI. The graphs represent the cellular fluorescence (_*Cell*_Fluorescence) and the nuclear-to-cytoplasmic (Nuc/Cyt) ratio showing a pronounced nuclear translocation of Slug. **C** qRT-PCR analysis of *SNAI1* mRNA expression levels in senescent MDA-MB-231 cells. **D** Representative images and quantification of the mesenchymal marker N-cadherin (green) in senescent MDA-MB-231 cells. The nucleus was counterstained with DAPI. The graphs represent the cellular fluorescence (_*Cell*_Fluorescence) and the number of foci per nucleus. **E** qRT-PCR analysis of N-cadherin (*CDH2)* mRNA expression levels in senescent MDA-MB-231 cells. **F** Representative images and quantification of the epithelial marker E-cadherin (green) in senescent MDA-MB-231 cells. The nucleus was counterstained with DAPI. The graphs represent the average fluorescence intensity per cell (_*Avg*_Fluorescence) and the nuclear-to-cytoplasmic (Nuc/Cyt) ratio showing a pronounced nuclear translocation of E-cadherin. **G** Western blot analysis of E-cadherin expression in senescent MDA-MB-231 cells showing a cleaved fragment of approximately 43 kDa. Lysates from MCF-7 cells were used as a positive control for full-length E-cadherin expression. Ponceau staining is shown as a loading control. The original blots are presented in Supplementary Fig. 4. **H** qRT-PCR analysis of E-cadherin (*CDH1)* mRNA expression levels in senescent MDA-MB-231 cells. **I** Western blot showing E-cadherin expression in nuclear fractions of senescent (Sen) MDA-MB-231 cells, but not in their proliferating (Pro) counterparts. γH2AX and Lamin B1 blots were used as controls for subcellular fractionation. The original blots are presented in Supplementary Fig. 5. **J** Western blot analysis of Zonula occludens-1 (ZO-1) expression in senescent MDA-MB-231. GAPDH blot was used as loading control. The original blots are presented in Supplementary Fig. 6. *p ≤ 0.05; ***p ≤ 0.001; ****p ≤ *0.0001*.

We first assessed vimentin, a key marker for EMT, that was significantly upregulated in these senescent MDA-MB-231 cells (Fig. 3A). These cells also showed an upregulation of the mesenchymal marker alpha-smooth muscle actin (α-SMA), whose expression is positively associated with invasive cancer phenotypes (Fig. 3A). In agreement with this upregulation of mesenchymal markers expression, *SNAI1* and *SNAI2* (Snail and Slug, respectively), two transcription factors that play a master role in regulating EMT, were also significantly upregulated upon etoposide treatment (Fig. 3B, C). Interestingly, immunofluorescence imaging and subsequent quantification revealed that Slug underwent nuclear translocation in senescent MDA-MB-231 cells (Fig. 3B), indicating its potential activation and involvement in senescence-associated EMT.

Cadherin-2, also known as Neural cadherin (N-cadherin), is a mesenchymal cadherin associated with EMT^50^. In our senescence model, the protein and mRNA levels of N-cadherin (*CDH2*) were significantly upregulated (Fig. 3D, E). Interestingly, immunofluorescence images indicated an increased nuclear localization of the protein that showed a nuclear foci pattern (Fig. 3D). The quantification of the number of foci per nucleus validated this observation and showed a significant upregulation of nuclear N-cadherin (Fig. 3D). This expression pattern of N-cadherin aligns with its role in EMT^51^, highlighting a function that is distinct from or extends beyond its traditional role as a cell adhesion molecule.

During EMT, the cell adhesion molecule E-cadherin is expected to get downregulated. Surprisingly, in our model, the levels of the key epithelial marker E-cadherin were not downregulated. In fact, immunofluorescence images show a significant increase in total E-cadherin levels (Fig. 3F). This upregulation was also confirmed by qRT-PCR, showing an increase in E-cadherin mRNA (*CDH1*) levels (Fig. 3H). Western blot analysis revealed the absence of full-length E-cadherin (~120 kDa) in MDA-MB-231 cells. However, a band of approximately 43 kDa appeared specifically in senescent cells, suggesting that proliferating MDA-MB-231 cells lack the membrane-bound protein, while senescent cells express a cleaved fragment or proteolytic product rather than the full-length E-cadherin (Fig. 3G). These findings are consistent with previous reports indicating that E-cadherin can undergo proteolytic cleavage, generating a cytosolic fragment capable of nuclear translocation through interaction with p120-catenin, a natural partner of β-catenin^52^. Interestingly, by investigating the subcellular localization of E-cadherin, we found that the protein was highly expressed in the nucleus of etoposide-induced senescent cells, which was further confirmed by the quantification of the nuclear-to-cytoplasmic ratio that is significantly increased in these senescent cells (Fig. 3F). These results were further confirmed by Western blot analysis of nuclear and cytosolic fractions from both proliferating and senescent cells; showing that the E-cadherin fragment was present in the nuclear fraction of senescent cells (Fig. 3I). These results, suggesting a nuclear translocation of the protein, correlate with previously reported internalization of E-cadherin that is caused by EMT, or loss of adhesion, therefore promoting migration^52–54^. Together, these findings suggest that etoposide-induced senescent MDA-MB-231 cells have a pronounced mesenchymal phenotype, compared to their corresponding proliferating cells. To further confirm the loss of cell-cell adhesion in senescent cells, we examined the expression of Zonula occludens-1 (ZO-1), a key protein associated with cell-cell contact integrity. Western blot analysis revealed a marked downregulation of ZO-1 in senescent MDA-MB-231 cells compared to their proliferating counterparts (Fig. 3J), further supporting the loss of intercellular adhesion associated with senescence.

### β-catenin pathway activation in etoposide-induced senescent MDA-MB-231 cells

**Fig. 4:**
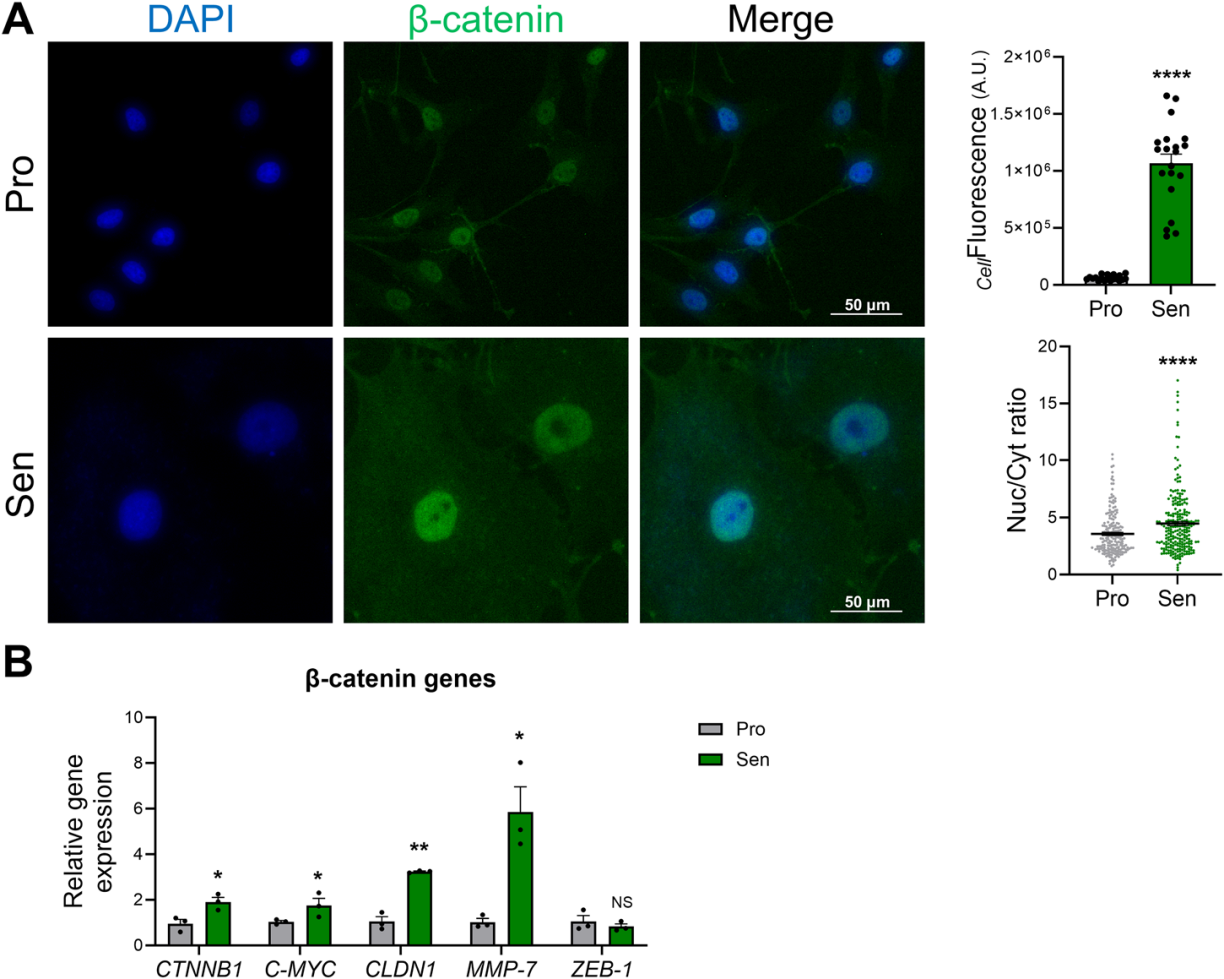
Etoposide promotes nuclear accumulation of β-catenin and activation of the Wnt/β-catenin signaling pathway in senescent MDA-MB-231 cells. **A** Representative images and quantitative analyses of β-catenin (green) expression. The nucleus was counterstained with DAPI. The graphs represent the cellular fluorescence (_*Cell*_Fluorescence) and the nuclear-to-cytoplasmic (Nuc/Cyt) ratio showing a pronounced nuclear translocation of β-catenin. **B** qRT-PCR analysis of β-catenin transcript (*CTNNB1*) and its target genes: *C-MYC*, *claudin-1* (*CLDN1*), *ZEB-1*, and *MMP-7* mRNA in senescent MDA-MB-231. NS: not significant; *p ≤ 0.05; **p ≤ 0.01; ****p ≤ *0.0001*.

Since β-catenin is a major player in EMT and is closely associated with E-cadherin at the plasma membrane level^55^, we next assessed whether etoposide treatment affects β-catenin expression profile in MDA-MB-231 cells. To this end, β-catenin levels and its subcellular localization were studied in etoposide-induced senescent MDA-MB-231 cells. β-catenin protein and mRNA levels were significantly higher in etoposide-induced senescent cells (Fig. 4A, B). Interestingly, immunofluorescence images show a nuclear presence of β-catenin in proliferating MDA-MB-231 cells (Fig. 4A), suggesting an active Wnt/β-catenin signaling that is known to be associated with various oncogenic processes including increased cell proliferation and EMT^56^. Upon senescence induction by etoposide treatment, an increased nuclear accumulation of β-catenin was observed, which was further confirmed by a significant increase in the nuclear-to-cytoplasmic ratio in senescent cells, compared to control proliferating cells (Fig. 4A). Since nuclear accumulation of β-catenin correlates with gene regulation, the expression of β-catenin-target genes was evaluated. Among these genes, *C-MYC*, *CLDN1*, and *MMP-7* were significantly upregulated in senescent cells (Fig. 4B), all known for their important role in EMT^57–59^. Despite the several functional links reported between ZEB-1 and β-catenin, *ZEB-1* mRNA levels were not affected by the pronounced β-catenin signaling (Fig. 4B). This, however, does not exclude the possibility of functional involvement, as ZEB-1 activity can be regulated post-transcriptionally^60,61^. Altogether, our results show that β-catenin signaling is accentuated in etoposide-induced senescent MDA-MB-231 cells and suggest that this enhanced activity promotes EMT by upregulating β-catenin target genes involved in this process, independently of additional ZEB-1 upregulation.

### Etoposide-induced senescence promotes migratory behavior in MDA-MB-231 cells

**Fig. 5:**
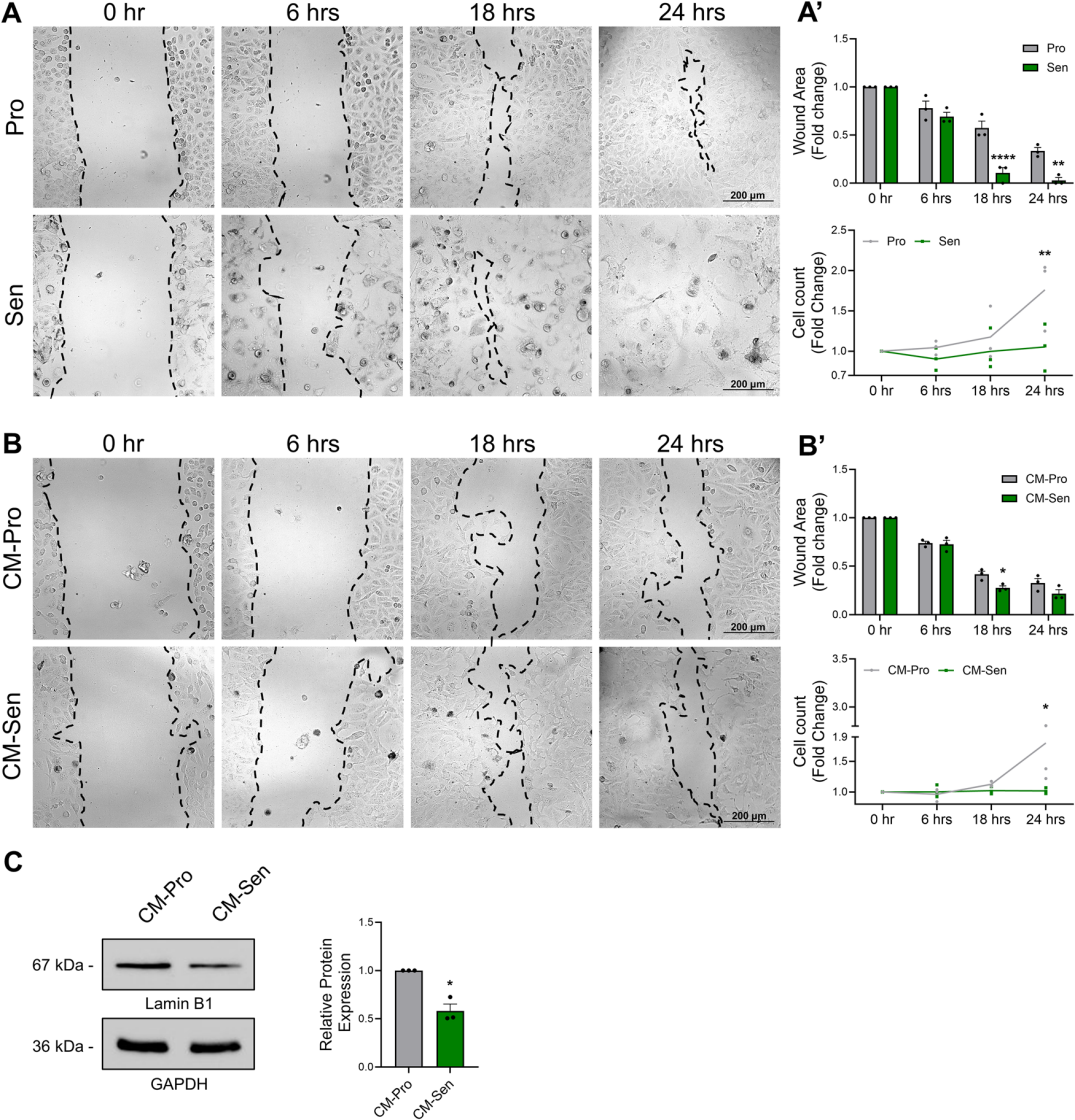
Etoposide-induced senescent MDA-MB-231 cells exhibit enhanced migratory capacity, which is transmitted to proliferating cells via their SASP. **A** Representative images from a scratch wound healing assay comparing proliferating (upper panel) and etoposide-treated senescent (lower panel) MDA-MB-231 cells. **A’** Quantification of the wound closure rate, reported as fold change relative to the initial wound area, along with the total number of cells per image. **B** Representative images from a cell migration assay showing MDA-MB-231 cells treated with conditioned media (CM) from proliferating control cells (CM-Pro) or from etoposide-induced senescent cells (CM-Sen). **B’** Quantification of the wound closure rate, reported as fold change relative to the initial wound area, along with the total number of cells per image. **C** Western blot analysis of Lamin B1 expression in MDA-MB-231 after treatment with CM from senescent MDA-MB-231. GAPDH blot was used as loading control. The original blots are presented in Supplementary Fig. 7. *p ≤ 0.05; **p ≤ 0.01; ****p ≤ *0.0001*.

Since mesenchymal phenotype is accompanied with increased migratory capacity^62^, we investigated the migration rate of these senescent MDA-MB-231 cells using wound healing scratch assay. To this end, on day 6 post-etoposide treatment, a wound was generated in both proliferative and senescent cells, and the area between the two wound borders was measured at different timepoints to estimate the migration speed of the cells. Our results show that the wound closure rate is higher in etoposide-induced senescent cells compared to their proliferating controls, as evidenced by the significantly reduced wound area observed starting 18 hrs after wound generation (Fig. 5A, A’). We also quantified the number of cells surrounding the wound to assess proliferation dynamics. In fact, our results show that only after 24 hrs of wound generation, the number of proliferating cells increased, while the number of senescent cells remained constant compared to the 0 hr condition (Fig. 5A’), suggesting that senescent cells exhibit increased migration compared to their proliferating MDA-MB-231 counterparts. To further explore these findings, conditioned media collected from senescent MDA-MB-231 cells (CM-Sen) was used to treat proliferating cells undergoing a scratch wound healing assay (Fig. 5B, B’). The wound area was then compared to that of cells treated with conditioned media from proliferating MDA-MB-231 cells (CM-Pro). At 18 hrs, the wound area was significantly smaller in the CM-Sen–treated condition compared to the CM-Pro condition, suggesting that the SASP-containing CM accelerated wound closure, with migration as a possible mechanism. However, by 24 hrs, the wound closure rate in the CM-Pro–treated cells had recovered and became comparable to that of the CM-Sen group (Fig. 5B, B’). This recovery was due to a significant increase in cell numbers under proliferative conditions compared to the 0 hr condition, as these cells continued to divide (Fig. 5B’). In contrast, the number of cells treated with CM-Sen remained stable over the different time points (Fig. 5B’). These findings suggest that CM-Sen–treated cells exhibit senescence features, as evidenced by the significant downregulation of Lamin B1 (Fig. 5C). Together, these findings demonstrate that etoposide-induced senescent MDA-MB-231 cells exhibit increased migratory capacity compared to their proliferating counterparts, consistent with the enhanced EMT observed following etoposide treatment. This phenotype can also be transmitted via the SASP secreted by senescent cells, promoting the migration of neighboring cells.

### Etoposide-induced senescent MDA-MB-231 cells express stemness markers

**Fig. 6:**
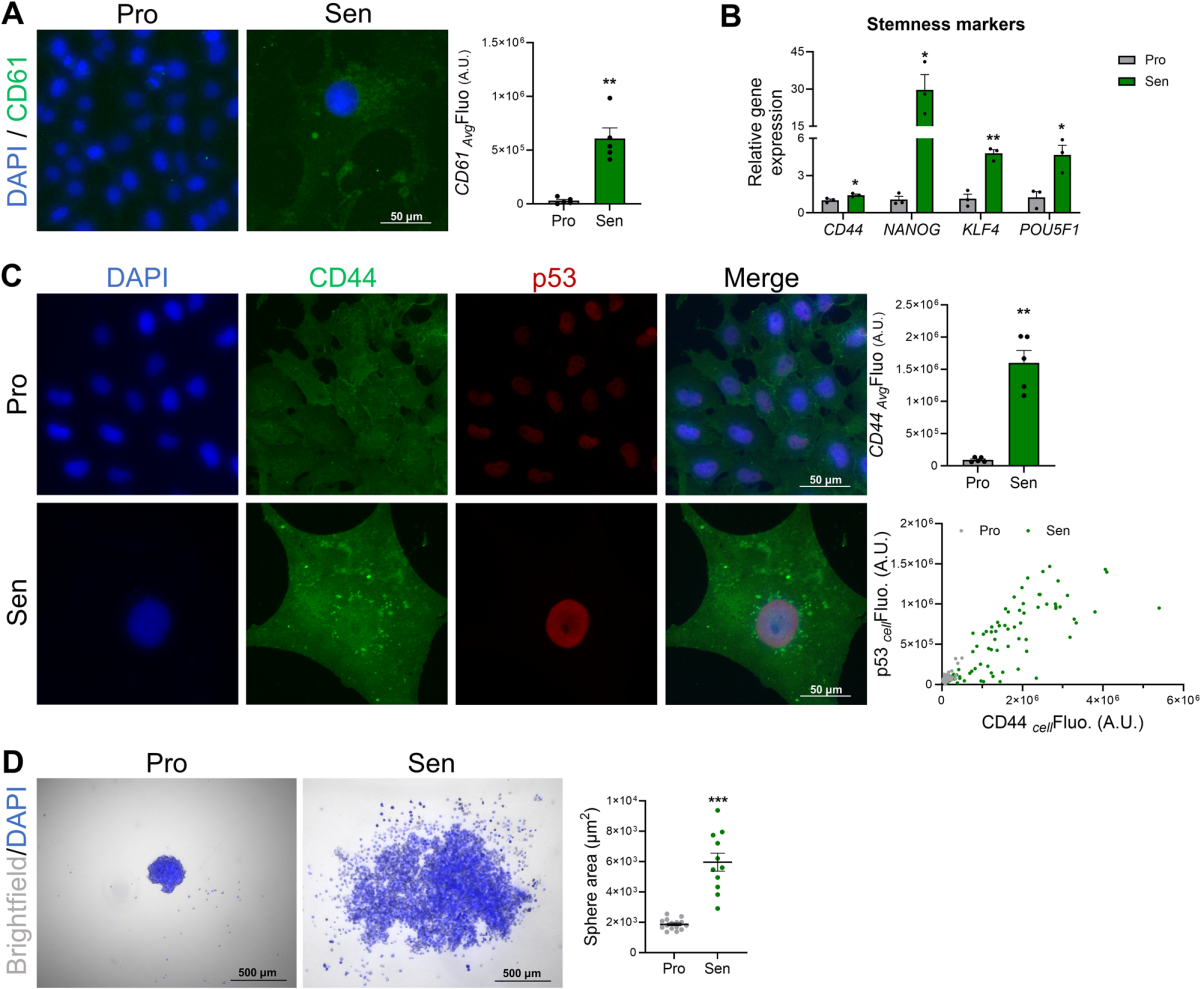
Stemness markers expression in senescent MDA-MB-231 cells. **A** Representative images and quantification of CD61 (green) expression in senescent MDA-MB-231 cells. The nucleus was counterstained with DAPI. The graphs represent the average fluorescence intensity per cell (_*Avg*_Fluorescence). **B** qRT-PCR analysis of stemness markers: *CD44*, *NANOG*, *KLF4*, and *POU5F1* mRNA in senescent MDA-MB-231 cells. **C** Representative images and quantification of double immunofluorescence showing CD44 (green) and p53 (red) expression in senescent MDA-MB-231 cells. The graphs represent the average fluorescence intensity per cell (_*Avg*_Fluorescence) of CD44. The scatter plot analysis of single-cell fluorescence intensities shows that etoposide-induced senescent MDA-MB-231 cells exhibit increased expression of both CD44 and p53 compared to proliferating cells. **D** Representative phase-contrast images show distinct morphological differences between tumor spheres derived from proliferating and senescent cells. Quantification of tumor sphere area confirmed a significant increase in sphere size variability and reduced compactness in senescent conditions compared to proliferating ones. *p ≤ 0.05; **p ≤ 0.01; ***p ≤ *0.001*.

Since EMT is strongly associated with cellular plasticity, we checked whether upon acquiring senescence phenotype, MDA-MB-231 express stem cell-like features. To this end, stemness markers were probed by immunofluorescence and qRT-PCR. Immunofluorescence images and their respective quantifications show that CD44 and CD61 expression increases significantly in senescent MDA-MB-231 cells compared to proliferating control cells (Fig. 6A, C). Other stemness markers were also significantly upregulated including *NANOG, KLF4*, and *POU5F1*, which are known as core stemness-associated transcription factors (Fig. 6B). Interestingly, double immunofluorescence showed that senescent cells expressing high levels of CD44 are also p53^+^ (Fig. 6C). In fact, senescent MDA-MB-231 cells show elevated expression of both p53 and CD44 compared to proliferating cells. A positive correlation between CD44 and p53 expression was also observed, particularly in senescent cells, indicating that cells with high CD44 levels tend to also express high levels of p53 (Fig. 6C). To evaluate the functional impact of stem-like features in senescent cells, we performed sphere formation assay in which senescent MDA-MB-231 cells were assessed for their ability to form spheres under non-adherent conditions, a readout of self-renewal and tumor-initiating potential. This assay revealed that senescent cells formed loose, irregular tumor spheres, in contrast to the compact, uniform spheres formed by proliferating cells (Fig. 6D). These observations are consistent with the reduced adhesion and enhanced mesenchymal traits observed in our senescent model. To rule out the possibility that the increase in sphere size was due to a higher cell number under senescent conditions, the spheres were dissociated and counted, and no significant difference in cell number was observed between conditions (data not shown). Together, these findings indicate that senescent MDA-MB-231 cells retain stem-like characteristics without re-entering the cell cycle by day 6 following etoposide treatment, and reveal potential reprogramming events occurring post-senescence, which support the link between senescence, EMT, and stemness.

### Blocking TGF-β and/or Wnt/β-catenin pathways downregulate profibrotic, mesenchymal, stemness features, and migration in senescent MDA-MB-231 cells

**Fig. 7:**
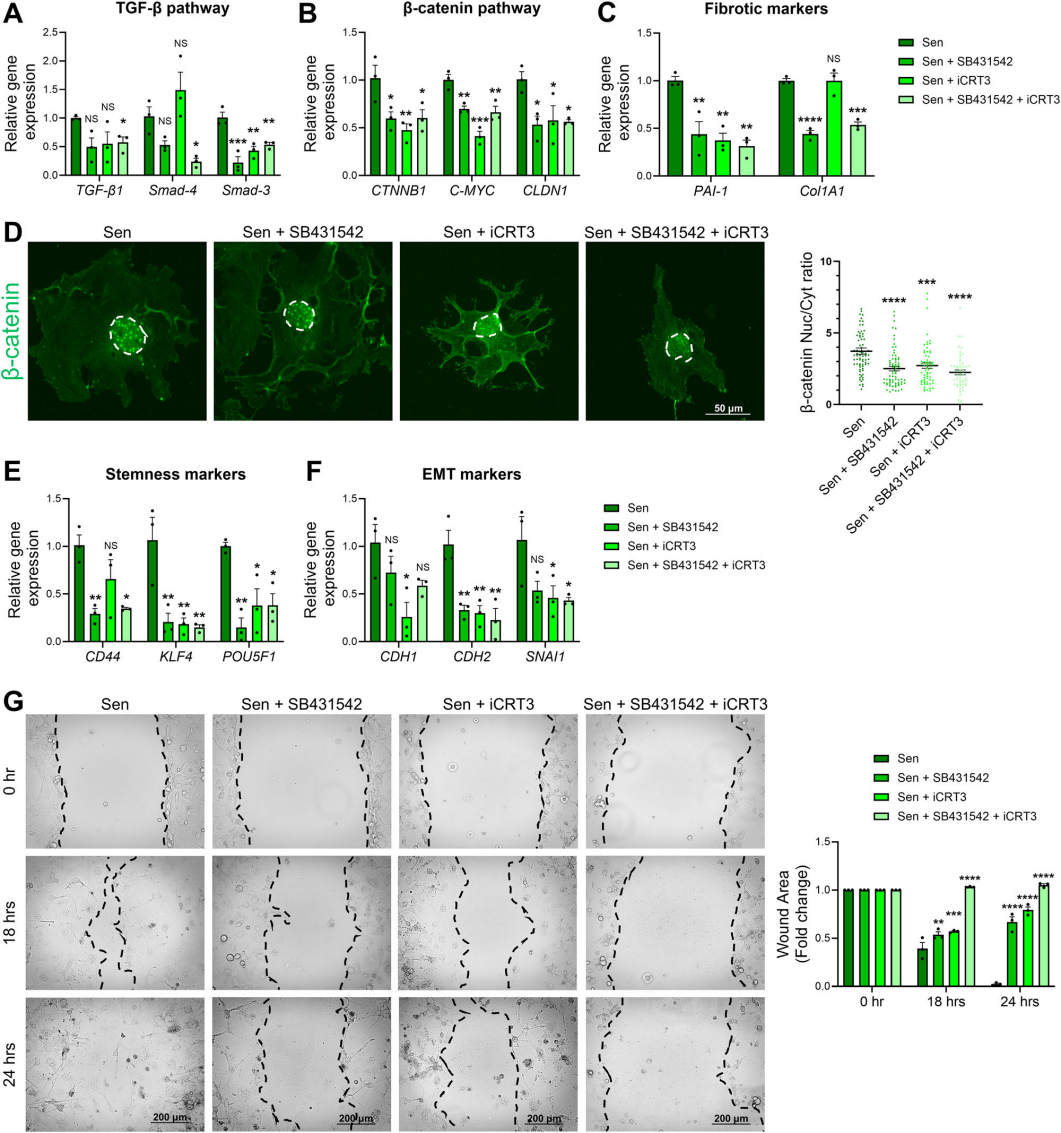
Downregulation of fibrosis, mesenchymal, and stemness markers following pharmacological inhibition of the TGF-β and Wnt/β-catenin pathways. qRT-PCR analysis of (**A**) TGF-β pathway, (**B**) β-catenin pathway, and (**C**) fibrotic markers in senescent MDA-MB-231 cells after blocking TGF-β and/or Wnt/β-catenin pathways using SB431542 and iCRT3, respectively. **D** Representative images and quantitative analyses of β-catenin (green) expression in senescent MDA-MB-231 cells after blocking TGF-β and/or Wnt/β-catenin pathways. The nucleus was counterstained with DAPI (not shown) and is outlined with dashed lines for visibility. The graph represents the nuclear-to-cytoplasmic (Nuc/Cyt) ratio showing a decreased nuclear translocation of β-catenin when TGF-β and/or Wnt/β-catenin pathways are inhibited. **E**, **F** qRT-PCR analysis of stemness and EMT markers in senescent MDA-MB-231 cells after blocking TGF-β and/or Wnt/β-catenin pathways. **G** Representative images from a scratch wound healing assay showing the migration of senescent MDA-MB-231 cells in the presence or absence of TGF-β and/or Wnt/β-catenin pathway inhibitors, SB431542 and iCRT3, respectively. The wound closure rate was quantified and expressed as a fold change relative to the initial wound area. NS: not significant; *p ≤ 0.05; **p ≤ 0.01; ***p ≤ 0.001; ****p ≤ *0.0001*.

To further validate that the activation of TGF-β and Wnt/β-catenin pathways in etoposide-induced senescent MDA-MB-231 cells is responsible for the observed phenotypes (fibrotic, mesenchymal, and stemness-like), these pathways were blocked pharmacologically using SB431542 and iCRT3, blockers of TGF-β and Wnt/β-catenin pathways, respectively. Then, mRNA levels of fibrosis, mesenchymal, and stemness markers were assessed using qRT-PCR. Blocking TGF-β pathway alone was able to significantly abolish the expression of *Smad-3* mRNA, with a trend to decrease *TGF-β1* and *Smad-4* expressions (Fig. 7A). Blocking Wnt/β-catenin pathway did not affect the expression of *Smad-4*, while *TGF-β1* tends to decrease, and *Smad-3* expression was significantly decreased when senescent cells were treated with iCRT3 (Fig. 7A). When treated with both inhibitors, *TGF-β1*, *Smad-3*, and *Smad-4* mRNA expression was significantly downregulated when compared to senescent control cells, suggesting an efficient inhibition of TGF-β pathway (Fig. 7A). Blocking these pathways, either individually or together, also significantly downregulated β-catenin (*CTNNB1*), *C-MYC*, and claudin-1 (*CLDN1*) mRNA levels (Fig. 7B), highlighting the crosstalk between the TGF-β and Wnt/β-catenin pathways in regulating the expression of these genes. Also, nuclear localization of β-catenin was significantly decreased when blocking these pathways, either individually or together (Fig. 7D), which may explain the decreased expression of β-catenin target genes. Together, our findings support the efficient pharmacological inhibition of both pathways.

Fibrotic markers’ expression was also evaluated after blocking TGF-β and/or Wnt/β-catenin pathways. Results showed a significant decrease in *PAI-1* expression in all conditions (Fig. 7C), while collagen-I mRNA levels were significantly downregulated when blocking TGF-β pathway alone or in combination with Wnt/β-catenin pathway (Fig. 7C), suggesting that fibrotic markers’ expression is also regulated by the crosstalk between TGF-β and Wnt/β-catenin pathways. CD44, known as stemness and mesenchymal marker, was significantly downregulated in senescent cells when treated with TGF-β inhibitor alone or in combination with Wnt/β-catenin inhibitor (Fig. 7E). *KLF4* and *POU5F1* mRNA levels were also significantly downregulated in senescent cells treated with the TGF-β inhibitor alone, the Wnt/β-catenin inhibitor alone, or combined (Fig. 7E). Together these findings suggest that stemness markers expression in senescent MDA-MB-231 cells is regulated by TGF-β and Wnt/β-catenin pathways. E-cadherin (*CDH1*), N-cadherin (*CDH2*), and *SNAI1* mRNA levels were also significantly downregulated when Wnt/β-catenin pathway was blocked (Fig. 7F). In addition, *CDH2* and *SNAI1* mRNA levels were significantly downregulated when both pathways were blocked. These findings suggest that EMT markers expression in senescent MDA-MB-231 cells is regulated by TGF-β and Wnt/β-catenin pathways. Despite the observed changes in the molecular profile of mesenchymal marker expression, we next investigated the functional consequences of blocking the TGF-β and Wnt/β-catenin pathways. To this end, a wound healing assay was performed in the presence of the pharmacological inhibitors targeting these pathways (Fig. 7G). Our results show that the rate of wound closure was markedly reduced under conditions where either TGF-β or Wnt/β-catenin signaling was inhibited, compared to untreated senescent controls. Interestingly, when both pathways were simultaneously inhibited, the wound area at 24 hrs remained comparable to that at 0 hr, suggesting a complete suppression of cell migration. These findings highlight the cooperative roles of the TGF-β and Wnt/β-catenin pathways in promoting the migratory capacity of senescent cells.

## Discussion

Despite its wide use in malignancy treatment, etoposide is poorly used in treating breast cancer^63^. In fact, clinical work testing etoposide in breast cancer treatment showed a poor response rate in the dose and schedules tested^63,64^. However, in vitro work highlighted the toxicity of etoposide on MCF-7 breast cancer cell lines^65^ and shed light on factors that may have a role in the development or progression of etoposide resistance^66^. Senescence is increasingly recognized as a contributing factor that drives chemotherapy resistance and promotes tumor heterogeneity^67^. In fact, chemotherapy-induced senescence is known to prevent the spread of cancer cells^15^. However, recent studies showed that these residual senescent cells can acquire pro-tumorigenic properties driving local and metastatic relapse^68^. These cells can also drive premature aging and age-related diseases^69–71^, highlighting the complex and multi-layered facets of cellular senescence in cancer biology. Interestingly, senescent-like phenotypes and senescence markers were detected within human breast cancer samples collected from patients who received chemotherapy^72^. In addition, several studies reported the expression of senescence markers in breast cancer cell lines exposed to chemotherapeutic drugs such as doxorubicin, etoposide, and cisplatin^16,33,34,73–79^. These studies converged in describing senescence induction as a new anticancer strategy and reporting the molecular mechanisms of the senescent phenotype observed in breast cancer cell lines. Doxorubicin- and cisplatin-induced senescence was fully elucidated in luminal MCF-7 and basal-like MDA-MB-231 triple-negative breast cancer cell lines. Etoposide-induced senescence has also been reported in MCF-7 and MDA-MB-231 cell lines; however, the cellular and molecular changes following therapy-induced senescence remain incompletely characterized. In this study, we show that a non-cytotoxic dose of etoposide (2.5 µM) induces senescence in MDA-MB-231 cells, an aggressive and highly chemoresistant breast cancer model. At the molecular level, markers of DNA damage response (γH2AX), cell cycle arrest (p53, *CDKN1A*, *CDKN2A*), and cell proliferation inhibition are all triggered upon etoposide treatment. In addition, increased SA-β-gal activity and downregulation of Lamin B1, along with SASP establishment, validate the senescent phenotype of the cells. Within the SASP, several pro-inflammatory cytokines, chemokines, and matrix-metalloproteases were upregulated. In our senescence model, GDF-15 and PDGF-α, two upregulated SASP components, are key signaling molecules of the TGF-β pathway that is known to be the master regulator of fibrosis^80–82^. Considering the well-established association between senescence, organ-specific fibrosis, and aging, we further evaluated the profibrotic potential of etoposide. Our senescence model shows a profibrogenic potential witnessed by an increase in collagen and fibronectin expression, suggesting an increase in ECM deposition, the core pathological process in fibrosis^83^. These findings are consistent with PDGF-α upregulation reported in this model, given that PDFG signaling is known to enhance ECM production and that TGF-β enhances PDGF signaling^84,85^. To further validate the activation of TGF-β signaling upon etoposide treatment, we described an upregulation of key mediators of the fibrotic response of TGF-β signaling including CTGF, CCN1, *Smad-3*, and *Smad-4* in this senescence model^47,86^. Together, these findings show that breast cancer cells exposed to a non-cytotoxic dose of etoposide activate TGF-β pathway and acquire profibrotic potential, which may translate into fibrocystic changes in breast tissue.

In addition to regulating numerous cellular processes, including cell growth, differentiation, immune responses, and tissue homeostasis^87^, TGF-β signaling plays a double-edged role in cancer. In early stages of cancer, TGF-β acts as a tumor suppressor by inhibiting cell proliferation and inducing apoptosis; while in advanced cancer, TGF-β promotes tumor progression by inducing EMT, enhancing cell migration, and suppressing anti-tumor immunity^88^. Since MDA-MB-231 cells are invasive mesenchymal-like cells, their mesenchymal and migratory properties were evaluated after etoposide treatment. Mesenchymal markers, such as vimentin and α-SMA, and the transcription factors Snail1 and Snail2, known as EMT-inducing transcription factors^89^, were significantly upregulated after etoposide treatment, suggesting a pronounced transition into the mesenchymal phenotype. Upregulation of N-cadherin followed by the downregulation of E-cadherin are also hallmarks of EMT resulting in the acquisition of an aggressive tumor phenotype^90^. In our model, N-cadherin was upregulated and, interestingly, nuclear N-cadherin accumulation was accentuated in etoposide-treated cells, indicating an ongoing EMT^90,91^. Surprisingly, E-cadherin was not downregulated in senescent MDA-MB-231 cells, as typically expected during EMT; however, a nuclear accumulation was observed following etoposide-induced senescence. In our study, full-length E-cadherin was not detected in MDA-MB-231 cells, consistent with previous reports describing the mesenchymal phenotype and low epithelial marker expression of this cell line^54,92–94^. Instead, we revealed the presence of a shorter E-cadherin fragment of approximately 43 kDa, predominantly observed in senescent cells. This truncated form likely represents a cytosolic fragment generated through proteolytic cleavage of the membrane-bound E-cadherin by metalloproteinases^52,95,96^ or other proteases activated during senescence. The detection of this fragment in the nucleus of senescent cells suggests a potential translocation of cleaved E-cadherin to the nucleus, consistent with previous findings showing that E-cadherin intracellular fragments can interact with p120-catenin, a natural partner of β-catenin^52^, to modulate gene expression and activate Wnt/β-catenin pathway signaling^96^. Such nuclear localization of E-cadherin fragments has been proposed to influence transcriptional regulation of genes involved in differentiation, adhesion, and EMT^96,97^. Therefore, our findings not only confirm the absence of full-length E-cadherin in MDA-MB-231 cells but also support a possible regulatory role of its cleaved fragment in the nuclear compartment during senescence. Of note, nuclear localization of E- and N-cadherins was also seen in proliferative MDA-MB-231 cells, which correlated with their mesenchymal-like state^51,98^. However, the accentuation of nuclear localization suggests that etoposide-induced senescence in MDA-MB-231 cells stresses their mesenchymal phenotype. In fact, upregulation and nuclear localization of E- and N-cadherins have been reported to enhance the invasion and migration of cancer cells by regulating EMT and stemness properties^51,95,98–100^. These findings align with our results showing an increased migratory capacity of senescent MDA-MB-231 cells that were able to close the wound, in a scratch wound healing assay, faster than proliferative cells. These results highlight that etoposide-induced senescence promotes increased cell motility, a key risk factor of metastasis and cancer recurrence. This effect is likely mediated by the SASP components produced by senescent cells that can act in a paracrine manner to remodel the tumor microenvironment and influence the behavior of neighboring non-senescent cells. In fact, conditioned media (CM) derived from etoposide-induced senescent cells significantly increases the migration of proliferative breast cancer cells. Key SASP components such as IL-6, IL-8, and MMPs are known to promote EMT, increase motility, and enhance invasiveness in cancer cells^101^. These components are significantly present in the SASP of etoposide-induced senescent MDA-MB-231 cells and may therefore contribute to the paracrine accentuation of the mesenchymal phenotype in proliferative cancer cells. These findings emphasize the role of the SASP in promoting cancer cell migration by creating a pro-inflammatory, pro-senescent, and pro-invasive tumor microenvironment.

Traditionally, N- and E-cadherins are transmembrane adhesion proteins that are localized at the plasma membrane. Their nuclear accumulation can be triggered by cellular stress, EMT, proteolytic cleavage by MMPs, and interaction with β-catenin^102–104^. In our model, cellular stress, ongoing EMT, and MMP overexpression are detectable; however, the state of β-catenin is not understood. E-cadherin is known to recruit β-catenin to the cell membrane and prevent its nuclear localization and transactivation^105^. In a previous study, N-cadherin nuclear localization was reported in colorectal cancer treated with lithium chloride, a chemical known as an activator of Wnt/β-catenin pathway^106^. Therefore, nuclear localization of N- and E-cadherins can suggest an activation of β-catenin signaling pathway in senescent MDA-MB-231 breast cancer cells. In our model, subcellular localization of β-catenin is comparable to that of E-cadherin in both proliferative and senescent MDA-MB-231 cells. In fact, β-catenin was mainly localized in the nucleus of proliferative cells, and this nuclear accumulation was accentuated in etoposide-treated senescent cells. In addition, a general upregulation of β-catenin expression levels was detectable in senescent MDA-MB-231 cells. Together, these findings suggest an activation of β-catenin pathway that was further confirmed by the upregulation of its target genes *C-MYC, CLDN1*, and *MMP-7*. These findings are consistent with the activated TGF-β signaling reported in our study, taking into account the crosstalk between TGF-β/Smad and Wnt/β-catenin signaling pathways and the crucial role of β-catenin downstream of TGF-β, which can also regulate EMT^107^.

The transcription factor Snail is known to confer cancer stem cell-like traits to tumor cells and promotes drug resistance, tumor recurrence, and metastasis^108^. In the senescence model developed in this study, Snail1 and Snail2 were significantly upregulated. In addition, senescent MDA-MB-231 cells showed a significant increase in stemness markers’ expression. These cells continued to express high levels of p53, further emphasizing that the acquisition of stem cell-like traits was occurring despite the cell cycle arrest characteristic of senescence. Interestingly, senescent MDA-MB-231 cells retained the ability to form tumor spheres, indicating that they preserved, at least partially, their self-renewal and tumor-initiating potential. However, the resulting spheres appeared irregular and loosely organized, in contrast to the compact and uniform spheres formed by proliferating cells. This morphological difference is consistent with the observed reduction in cell-cell adhesion and the enhanced mesenchymal features characterizing the senescent state. These findings suggest that, although senescence dampens proliferative capacity, certain stem-like and plasticity-associated traits may persist, potentially contributing to tumor heterogeneity and therapy resistance. However, whether these cells will re-enter the cell cycle and resume proliferation with time remains unknown, and this possibility may underlie cancer recurrence. In fact, cellular senescence can paradoxically promote cancer stemness and tumor aggressiveness, leading to cancer relapse and metastasis, particularly through Wnt/β-catenin pathway activation, and can generate tumor-initiating cells^109^. Since all these features are detected in etoposide-induced senescent MDA-MB-231 cells, our results may explain the poor clinical outcomes of etoposide use in breast cancer management.

TGF-β/Smad signaling is known to induce EMT, which correlates with stemness and invasiveness in cancer cells^110–112^, and is also recognized as the master regulator of tissue fibrosis^113^. Sustained activation of Wnt/β-catenin pathway is not only associated with fibrogenesis and aging-related tissue fibrosis^114,115^, but also required for stemness markers expression^116–118^, and the progression of TGF-β-mediated fibrosis^119^. Therefore, the two pathways engage in crosstalk that plays an essential role in activating the genetic machinery initiating profibrotic changes^120,121^ and cancer progression^122,123^. However, whether this crosstalk occurs in senescent cells and influences senescence-related downstream responses to chemotherapy remains unexplored. Since both pathways are activated in our etoposide-induced senescent MDA-MB-231 cells, we further explored their role in the observed features of senescent MDA-MB-231 cells including fibrogenic response, acquisition of stemness traits, and enhanced EMT. Our findings indicate that the crosstalk between the two pathways is essential for maintaining these features in senescent cells, as pharmacological inhibition of either pathway, or both, markedly reduces the expression of fibrotic, stemness, and mesenchymal markers, and significantly impairs cellular migration. While TGF-β and Wnt/β-catenin signaling pathways are known to cooperate in promoting fibrosis in non-cancerous tissues, our findings are the first to outline their crucial role in reinforcing senescence-associated phenotypes in cancer cells, while simultaneously promoting features commonly linked to tumor progression and resistance, including cellular plasticity, stemness, and EMT. It is important to note that the results from pharmacological inhibition and functional readouts could be further complemented by genetic perturbation of key components of the TGF-β and Wnt/β-catenin pathways (e.g., *CTNNB1*, *SMAD3*, or *TGFBR1* knockdown) to provide more comprehensive mechanistic insights into their roles in the observed features of senescent MDA-MB-231 cells. These findings suggest that inhibition of TGF-β/Wnt/β-catenin crosstalk may be a promising strategy for alleviating chemotherapy-induced side effects associated with the accumulation of senescent cells. Notably, this therapeutic approach has been previously explored in the context of idiopathic pulmonary fibrosis, a condition also characterized by the accumulation of senescent cells, and demonstrated promising anti-fibrotic potential^124^.

**Fig. 8:**
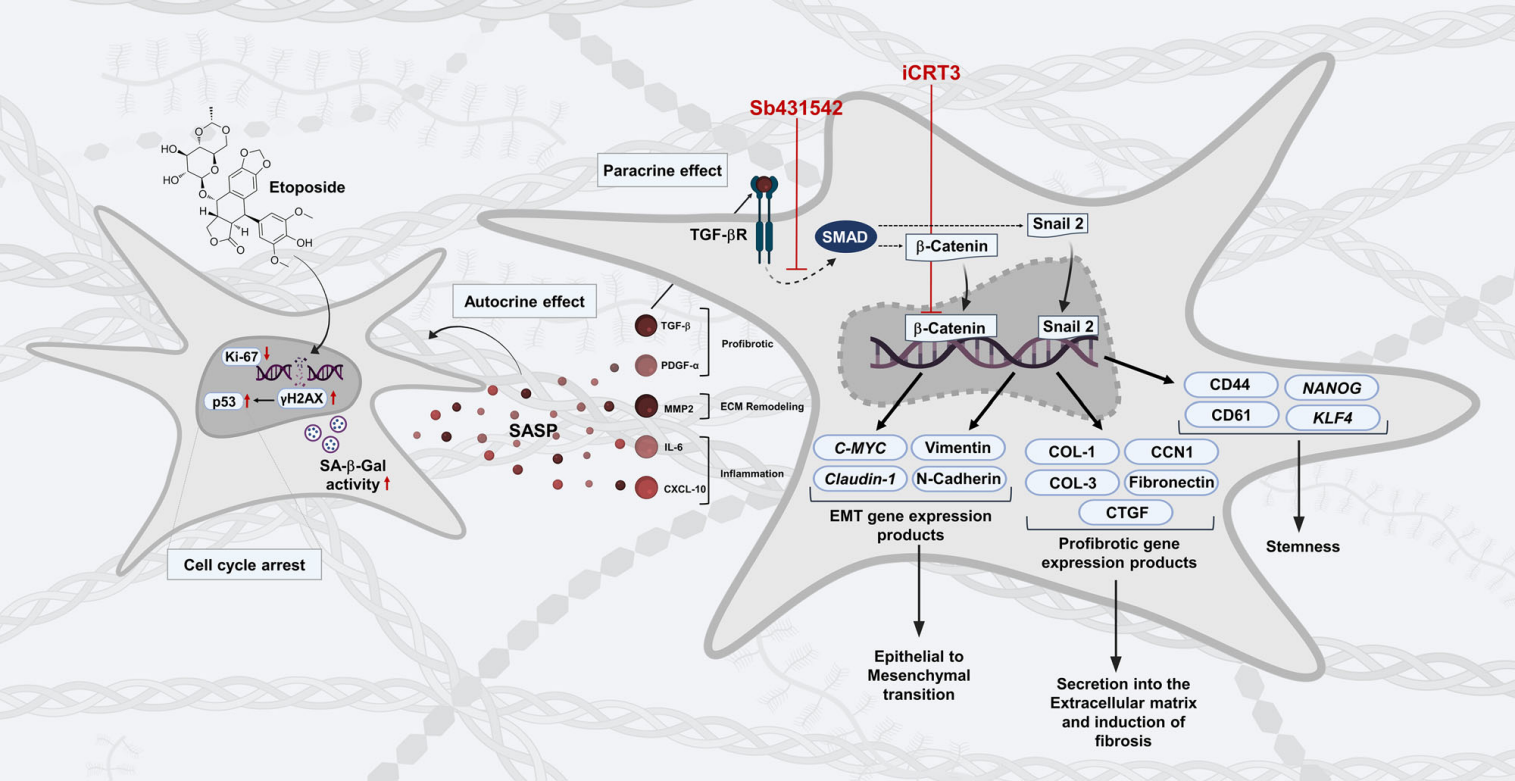
Mechanistic insights into molecular and cellular changes induced by etoposide-mediated senescence in MDA-MB-231 cells. Low-dose etoposide, a topoisomerase II inhibitor, induces DNA damage and cell cycle arrest, leading to cellular senescence. Senescent cells secrete a senescence-associated secretory phenotype (SASP), with key activators of the TGF-β pathway. TGF-β activation crosstalks with Wnt/β-catenin signaling, promoting profibrotic responses, epithelial-mesenchymal transition (EMT), and the expression of stemness markers. Functionally, these molecular changes drive extracellular matrix (ECM) remodeling, enhance cell migration, reduce adhesion, and maintain cancer stem cell properties, increasing cellular plasticity and stem-like traits.

Overall, our study demonstrates that low-dose etoposide induces a stable senescence program in MDA-MB-231 breast cancer cells, characterized by a SASP enriched in inflammatory, EMT-related, and profibrotic factors (Fig. 8). Functionally, these senescent cells exhibit an accentuated mesenchymal phenotype, enhanced migratory capacity, and acquisition of stem-like traits while maintaining cell cycle arrest. Together, these features may contribute to the formation of a pro-tumorigenic and chemoresistant microenvironment, marked by chronic inflammation, increased cell migration, and fibrosis, which may elevate the risk of tumor progression and cancer recurrence. Mechanistically, we show that interconnected TGF-β and Wnt/β-catenin signaling pathways are activated in these senescent cells and cooperatively drive their profibrotic response, EMT, and induction of stemness markers (Fig. 8). This study provides the first evidence that crosstalk between these pathways underlies plasticity-associated features in senescent MDA-MB-231 cells. By elucidating the molecular and functional consequences of etoposide-induced senescence in an in vitro breast cancer model, our work offers a foundation for further investigations into whether senescence-associated EMT, stemness, and fibrosis are common outcomes of other cancer therapies. Such insights may ultimately help refine treatment strategies and support the development of senolytics as potential adjuvants to chemotherapy.

## Methods

### Cell culture

Breast cancer cell line (MDA-MB-231, MCF-7) were acquired from the American Type Culture Collection (ATCC). Cells were cultured in complete cell culture media containing Dulbecco’s modified Eagle medium/Nutrient Mixture F-12 (DMEM/F-12) or DMEM Dulbecco Modification of Eagle’s Medium with 4.5 g/L glucose, L-glutamine, and sodium pyruvate (Corning, 10-013-CV), supplemented with 10% fetal bovine serum (FBS), penicillin (100 units/mL) and streptomycin (10 µg/mL) and maintained at 37 °C in a humidified environment with 5% CO_2_.

### Etoposide treatment

Senescence was induced using non-cytotoxic concentrations of etoposide. Briefly, 5 × 10^5^ cells were seeded in 10 cm petri dishes and allowed to adhere for 24 hrs. The cells were then treated for 48 hrs with 2.5 µM etoposide (MedChemExpress) or an equivalent volume of DMSO (control), added to the complete culture medium. After 48 hrs, the culture medium was completely removed, and the cells were processed according to their downstream application. For immunofluorescence and senescence-associated beta-galactosidase (SA-β-gal) experiments, cells were harvested from the petri dishes using a standard trypsinization protocol, counted, and seeded at a density of 2 ×10^4^ cells (treated) and 2 × 10^3^ cells (control) onto glass coverslips in 24-well plates. For western blot and qRT-PCR experiments, cells were maintained in petri dishes for an additional 4 days, and the culture medium was replaced every 2 days. On day 6 post-treatment, cells grown on coverslips were fixed as described in the “Immunofluorescence” section, while cells cultured in petri dishes were collected using the trypsinization protocol and stored as dry pellets at -80 °C. Accordingly, all downstream analyses, including immunofluorescence, western blotting, and qRT-PCR, were performed on day 6 after etoposide treatment.

### Conditioned media collection

On day 3 post-treatment, MDA-MB-231 cells were seeded into 35 mm petri dishes at a density of 2.5 × 10^4^ cells for proliferating cells and 2 × 10^5^ cells for etoposide-treated cells in 2 mL of complete medium. On day 6 post-treatment, the culture media from both proliferating and senescent cells (CM-Ctrl or CM-Sen) were collected, centrifuged at 1000 g for 5 min at 4 °C to pellet debris, and the supernatant was used freshly for wound healing experiments.

### TGF-β and Wnt/β-catenin inhibitors assay

MDA-MB-231 cells were seeded in 6-well plates at a density of 10^5^ cells per well and allowed to adhere overnight. The following day, cells were treated with 2.5 µM etoposide alone, or in combination with one of the following conditions: 10 µM TGF-β inhibitor (SB431542, AdooQ Bioscience), 25 µM Wnt/β-catenin pathway inhibitor (iCRT3, AdooQ Bioscience), or a mixture of both inhibitors. On day 2 post-treatment, the media was removed and replaced daily with fresh medium containing the respective inhibitors to maintain pathways inhibition. On day 4, cells were detached using the standard trypsinization protocol, and cells were collected as dry pellets for subsequent qRT-PCR or seeded on coverslips for immunofluorescence.

### Cell proliferation assay

To assess cell proliferation, MDA-MB-231 cells were seeded in 6-well plates at a density of 2 × 10^5^ cells per well in triplicate. After allowing cells to adhere overnight, treatments were administered as described in the “Etoposide treatment” section. Cell proliferation was evaluated by collecting and counting cells on day 1, 2, 3, 5, and 6 post-treatment.

### Immunofluorescence

On day 6 post-treatment, cells on coverslips were washed once with phosphate-buffered saline (PBS) and fixed with 4% paraformaldehyde (PFA) in PBS for 10 min at room temperature (RT). Following fixation, cells were permeabilized with 0.2% Triton X-100 in PBS for 10 min. Subsequently, cells were blocked for 30 min in 3% bovine serum albumin (BSA) in PBST (PBS with 0.1% Tween 20). Cells were then incubated for 1.5 hr at room temperature (RT) in 1% BSA/PBST containing primary antibodies (Supplementary Table 1). After three washes with PBS, the cells were incubated for 45 min at RT with either CoraLite488-conjugated goat anti-rabbit IgG (SA00013-2, Proteintech) or Cy3-conjugated goat anti-mouse IgG (SA00009-2, Proteintech) at a dilution of 1:500 in 1% BSA/PBST. After the final washes, the coverslips were mounted using glycerol antifade mounting medium with DAPI (ab188804, Abcam) to stain DNA. The slides were then sealed and allowed to dry overnight before imaging with a ZOE™ Fluorescent Cell Imager.

For double immunofluorescence, cells were incubated simultaneously with the primary antibodies, washed with PBS, then incubated sequentially with each secondary antibody, with a series of three PBS washes between incubations. Finally, cells were washed and mounted as described previously.

For the decellularization experiment, coverslips were treated with 20 mM NH_4_OH for 5 min at RT, washed with PBS, and fixed with 4% PFA in PBS for 10 min at RT. Following fixation, cells were blocked and processed according to the classical immunofluorescence protocol described above.

### Image analysis and quantifications

All immunofluorescence quantifications were performed using ImageJ. For pan-nuclear staining, quantification of nuclear protein levels was performed as previously described^125^. Briefly, individual nuclei were outlined using the “Analyze Particles” tool in ImageJ, as previously described^126^. The area and integrated density of each nucleus were measured, along with several adjacent background readings. Nuclear fluorescence was calculated using the formula: _*Nuc*_Fluorescence = integrated density − (area of nucleus x mean fluorescence of background readings). For pan-cellular staining, the same procedure was applied to individual cells outlined manually, and the results were reported as “_*Cell*_Fluorescence”. Alternatively, the integrated density of the entire image was measured, along with several adjacent background readings, to calculate the average fluorescence (_*AVG*_Fluorescence) per cell using the formula: _*AVG*_Fluorescence = [integrated density − (area of the image x mean fluorescence of background readings)] ÷ number of cells per image. Notably, both quantification methods produced consistent results.

For foci staining, individual nuclei were outlined using the “Analyze Particles” tool in ImageJ, as previously described^126^. Within each ROI, foci were detected and counted using the “Find Maxima” tool, following the procedure outlined in^126^.

For nuclear-to-cytoplasmic ratio measurements, circular regions of interest (ROIs) were manually drawn in the nucleus and cytoplasm. For each ROI, corrected fluorescence was calculated as: corrected fluorescence = integrated density − (area x mean fluorescence of background readings). The nuclear-to-cytoplasmic ratio for each cell was determined by dividing the corrected nuclear fluorescence by the corrected cytoplasmic fluorescence.

### Cell migration assay

Cell migration was measured using would healing scratch assay. On day 3 post-treatment with etoposide, MDA-MB-231 cells were seeded into 35 mm petri dishes at a density of 2.5 × 10^4^ cells for proliferating cells and 2 × 10^5^ cells for treated cells. On day 6 post-treatment, a wound was generated in the monolayer using 200 µL pipette tip, the media was removed, and the dishes were washed to remove detached cells. The cells were fed with fresh media, and wound closure was monitored over time. Multiple images of the wound area were taken with a ZOE™ Fluorescent Cell Imager at 0, 6, 18, and 24 hrs. The wound area was measured and quantified using ImageJ software.

For the CM-mediated wound healing experiment, proliferating MDA-MB-231 cells were seeded as previously described. Following wound generation, cells were treated with either CM-Ctrl or CM-Sen, and wound closure was monitored and imaged using a ZOE™ Fluorescent Cell Imager. The wound area was quantified using ImageJ. Of note, at the end of the CM-mediated wound healing experiment, cells were detached using standard trypsinization protocol, then collected as dry pellets for subsequent western blot analysis.

For the wound healing experiment with inhibitors, cells were seeded and treated as described in the “TGF-β and Wnt/β-catenin inhibitors assay” section. On day 3, a wound was generated in the monolayer using a 200 µL pipette tip. The medium was then removed and the dishes were washed to eliminate any detached cells. Fresh medium containing the inhibitors was added, and wound closure was monitored and imaged using the ZOE™ Fluorescent Cell Imager. The wound area was measured and quantified using ImageJ software.

### Protein extraction and subcellular fractionation

Total proteins were extracted from frozen cell dry pellets using RIPA buffer supplemented with Protease Inhibitor Cocktail III (22020008, bioPLUS™). Cells were incubated in lysis buffer on ice for 15 min. Samples were then centrifuged at 14,000 g for 15 min at 4 °C. The supernatant was transferred to Eppendorf tubes and protein content was determined by BCA protein quantification kit (PO11, ABP Biosciences).

Subcellular fractionation was performed as previously described by Rima et al.^127^ with slight modifications. Cell pellets from proliferating and senescent cells were resuspended in a hypotonic lysis buffer containing 10 mM HEPES (pH 7.9), 10 mM KCl, 1.5 mM MgCl_2_, 0.5 mM dithiothreitol (DTT), and Protease Inhibitor Cocktail III (22020008, bioPLUS™), and incubated on ice for 5 min. Cells were mechanically disrupted using a pre-chilled Dounce homogenizer and centrifuged for 5 min at 200 g and 4 °C. The supernatant was retained as the cytoplasmic fraction, and the pellet was resuspended in RIPA buffer supplemented with Protease Inhibitor Cocktail III. The mixture was incubated on ice for 15 min, with gentle vortexing every 5 min. The suspension was then sonicated on ice (6 ×10 s pulses) and centrifuged at 14,000 g for 10 min at 4 °C. The resulting supernatant was collected as the nuclear fraction. Protein concentrations in both fractions were determined using the BCA Protein Quantification Kit (PO11, ABP Biosciences).

### Western blot

Protein extracts (10-15 µg per sample) were separated by SDS-PAGE on 10% or 12% resolving gels. Separated proteins were then electro-transferred to 0.2% nitrocellulose membrane (Biorad). The membrane was blocked in 5% milk in PBS containing 0.1% Tween for 30 min at 37 °C and then incubated for 2 hrs at 37 °C with the primary antibody (Supplementary Table 2). After 3 × 5 min washes with PBS containing 0.1% Tween, blots were incubated with 1:10000 HRP-conjugated anti-mouse or HRP-conjugated anti-rabbit (ProteinTech, SA00001-8 or SA00001-2) at 37 °C for 45 min. Bands were then visualized after incubation with Clarity™ Western ECL Substrate using ChemiDoc™ Touch Gel Imaging System (Biorad). Bands’ intensities were quantified using ImageJ and normalized to the loading control (α-tubulin or GAPDH).

### SA-β-galactosidase staining

SA-β-gal activity was detected using the Senescence Detection Kit (ab65351) according to the manufacturer’s instructions. Briefly, at the desired time points, cells were fixed with the provided fixative solution for 10 min at room temperature, followed by three PBS washes. Subsequently, cells were incubated in the staining solution overnight at 37 °C and observed under an inverted microscope for the development of the blue color. After staining, cells were washed three more times with PBS and the coverslips were mounted using a glycerol mounting medium. Finally, the slides were sealed and imaged using a Zeiss Axio Observer 7 microscope.

### RNA isolation, cDNA synthesis, and qRT-PCR

RNA was extracted from frozen cell pellets using the Macherey-Nagel™ NucleoSpin™ RNA Mini Kit, following the manufacturer’s instructions. RNA concentration and sample purity were measured using the NanoDrop ND-1000 spectrophotometer (Thermo Fisher). cDNA was synthesized from 250 to 500 ng of RNA using the FireScript RT cDNA Synthesis Mix (06-20-00100, Solis BioDyne) according to the manufacturer’s recommendations. The cDNA samples were diluted at a ratio of 1:10 and stored at -20 °C until use. Quantitative PCR was performed on a CFX96 Real-Time PCR Detection System (Bio-Rad) using iTaq™ Universal SYBR^®^ Green Supermix (Bio-Rad), following the manufacturer’s instructions. Briefly, cDNA samples, primers (Supplementary Table 3), and SYBR Green supermix were added to each well of the PCR plate. The PCR program included an initial cycle at 95 °C for 30 s, followed by 40 cycles of 95°C for 5 s and 60 °C for 30 s, with a final melt curve step. Cycle threshold (CT) values were exported and melt curves were analyzed using CFX Maestro™ Software. Relative gene expression was calculated using the ΔΔCT method with *RPLP0 (RPPO)* as the housekeeping gene. Results were expressed as fold change relative to the control condition.

### Annexin V/PI staining by flow cytometry

Annexin V/PI assay was performed according to the manufacturer’s instructions for the FITC Annexin V and PI Apoptosis Kit (ABP Biosciences, A026). Briefly, on day 2 post-etoposide treatment, cells were detached using standard trypsinization and resuspended in 50 µL of 1x binding buffer, followed by the addition of 2.5 µL FITC–Annexin V and 1 µL PI. Samples were incubated for 10 min in the dark, after which 200 µL of 1x binding buffer was added. A total of 150 µL of the stained cell suspension was transferred into a 96-well plate for analysis using a Guava easyCyte™ flow cytometer.

### 3D spheroid formation assay

To assess cancer stem cell properties, cells were allowed to form 3D spheroids using the hanging drop method as previously described^128^. Briefly, on day 2 post-etoposide treatment, both treated and control (proliferating) MDA-MB-231 cells were detached, counted, and resuspended in complete medium at a density of 2.5 × 10^5^ cells/mL. Four replicates of 10 µL droplets were pipetted onto the inverted lids of Corning 35 mm tissue culture-treated dishes filled with 2 mL of PBS to maintain humidity. The lids were then carefully re-inverted, placed back onto the PBS-filled dishes, and incubated at 37 °C for an additional 4 days. On day 6, spheroids were stained with DAPI, imaged using the EVOS M3000 imaging system, and their areas were quantified using ImageJ software.

### Statistics

Experiments were performed in at least triplicate, and data are presented as mean ± SEM; individual data points are also shown. A Student’s *t*-test was used to calculate the statistical significance of differences between two sets of averaged data. Statistical analysis of the qRT-PCR experiments with inhibitors was performed using one-way ANOVA, whereas wound healing and cell counting experiments were analyzed using two-way ANOVA. ANOVA was followed by Dunnett’s post hoc test for multiple comparisons, with the control group set as reference. A *p*-value ≤ *0.05* was considered statistically significant.

## Supplementary information

Supplementary Information

## Data Availability

The original results presented in the study are included in the article/supplementary data; further inquiries can be directed to the corresponding author.
