# Supplementary material for: Crosstalk between TGF-β and Wnt/β-catenin signaling drives fibrogenic and stem-like phenotypes in senescent MDA-MB-231 breast cancer cells
Source: NPJ Aging. 2026 Jan 3;12(1):22. doi: 10.1038/s41514-025-00322-0 (PMC12868629; doi:10.1038/s41514-025-00322-0)
Supplement: Supplementary file 1 — Supplementary Information [file 41514_2025_322_MOESM1_ESM.pdf]

Supplementary Table 1. List of Antibodies used for immunofluorescence experiments.

Antibody	Supplier	Reference	Dilution	Species
**Collagen 1**	Bio-Rad	2150-0001	1/100	Mouse
**Fibronectin**	Sino Biological	10314-T54	1/500	Rabbit
**Ki67**	Sino Biological	13180-MM01	1/500	Rabbit
**Collagen III**	Bio-Rad	2150-0100	1/500	Mouse
**CTGF**	Proteintech	25474-1-AP	1/500	Rabbit
**CYR61/CCN1**	proteintech	26689-1-AP	1/500	Rabbit
**P53**	ProteinTech	60283-2-Ig	1/1000	Rabbit
**Phospho-Histone H2A.X (Ser139)**	ProteinTech	29380-1-AP	1/1000	Rabbit
**Alpha smooth muscle actin**	ProteinTech	14395-1-AP	1/500	Rabbit
**Vimentin**	ProteinTech	10366-1-AP	1/500	Rabbit
**N-cadherin**	ProteinTech	22018-1-AP	1/500	Rabbit
**E-cadherin**	ProteinTech	20874-1-AP	1/500	Rabbit
**SNAI2/SLUG**	ProteinTech	12129-1-AP	1/500	Rabbit
**Beta Catenin**	ProteinTech	51067-2-AP	1/500	Rabbit
**CD61**	ProteinTech	18309-1-AP	1/250	Rabbit
**CD44**	ProteinTech	15675-1-AP	1/250	Rabbit

Supplementary Table 2. List of Antibodies used for western blot experiments.

Antibody	Supplier	Reference	Dilution	Species
**Lamin B1**	ProteinTech	12987-1-AP	1/5000	Rabbit
**E-cadherin**	ProteinTech	20874-1-AP	1/2000	Rabbit
**Zo-1**	ProteinTech	21773-1-AP	1/5000	Rabbit
**Phospho-Histone H2A.X (Ser139)**	ProteinTech	29380-1-AP	1/1000	Rabbit
**GAPDH**	ProteinTech	10494-1-AP	1/5000	Rabbit
**Alpha tubulin**	ThermoFisher	62204	1/5000	Mouse

Supplementary Table 3. List of primers used for qRT-PCR experiments.

Gene	Forward	Reverse
MMP-2	CGCTCAGATCCGTGGTGAG	TGTCACGTGGCGTCACAGT
MMP-9	CCCTGGAGACCTGAGAACCA	CCCGAGTGTAACCATAGCGG
CLDN1	ATGAGGATGGCTGTCATTGG	ATTGACTGGGGTCATAGGGT
MMP-7	TCGGAGGAGATGCTCACTTCGA	GGATCAGAGGAATGTCCCATACC
β-catenin (CTNNB1)	CACAAGCAGAGTGCTGAAGGTG	GATTCCTGAGAGTCCAAAGACAG
CDH1	GCCTCCTGAAAAGAGAGTGGAAG	TGGCAGTGTCTCTCCAAATCCG
CDH2	ATTGGACCATCACTCGGCTTA	CACACTGGCAAACCTTCACG
C-MYC	TCAAGAGGCGAACACACAAC	GGCCTTTTCATTGTTTTCCA
ZEB-1	ACCCTTGAAAGTGATCCAGC	CATTCCATTTTCTGTCTTCCGC
SNAIL1 (SNAI1)	ACAAGCACCAAGAGTCCG	ATG GCAGTGAGA AGGATGTG
RPPO	TTCATTGTGGGAGCAGAC	CAGCAGTTTCTCCAGAGC
IL-1α	GGTTGAGTTTAAGCCAATCCA	TGCTGACCTAGGCTTGATGA
IL-1β	TTCTTCGACACATGGGATAACG	TCCCGGAGCGTGCAGTTCA
IL-8	AAGGAAAACTGGGTGCAGAG	ATTGCATCTGGCAACCCTAC
CXCL-10	CCAGAATCGAAGGCCATCAA	CATTTCCTTGCTAACTGCTTTCAG
GDF-15	GGCCAACCAGAGCTGGGAAG	GCCCGAGAGATACGCAGGTG
IL-6	GCAGAAAAGGCAAAGAATC	CTACATTTGCCGAAGAGC
PDGF-α	GCAAGACCAGGACGGTCATTT	GGCACTTGACACTGCTCGT
COL1A1	GATTCCCTGGACCTAAAGGTGC	AGCCTCTCCATCTTTGCCAGCA
COL3A	TGGTCTGCAAGGAATGCCTGGA	TCTTTCCCTGGGACACCATCAG
PAI-1	AGCTCCTTGTACAGATGCCG	ACAACAGGAGGAGAAACCCA
TGF-β1	CAGAAATACAGCAACAATTCC	CTGAAGCAATAGTTGGTGTC
Smad-3	TGAGGCTGTCTACCAGTTGACC	GTGAGGACCTTGTCAAGCCACT
Smad-4	CTACCAGCACTGCCAACTTTCC	CCTGATGCTATCTGCAACAGTCC
CD44	AGACGAAGACAGTCCCTGGATCAC	TGTGTTTGCTCCACCTTCTTGACTC
NANOG	AAGGTCCCGGTCAAGAAACAG	CTTCTGCGTCACACCATTGC
KLF4	GGGAGAAGACACTGCGTCAA	TCCAGGTCCAGGAGATCGTT
POU5f1	GGTGGAGAGCAACTCCCGATG	CCAGGGTGATCCTCTTCTGC
CDKN2A	CGAATAGTTACGGTCGGAGG	TGAGAGTGGCGGGGTCG
CDKN1A	TCCTTTCCCTTCAGTACCCTCTC	CCTTCTTCTTGTGTGTCCCTTCC

Annexin V /PI staining by flow-cytometry for control and etoposide-treated MDA-MB-231 cells 48 hrs post-treatment

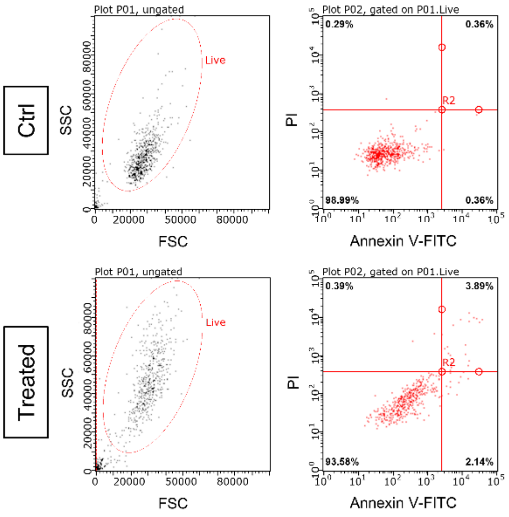

Suppl. Figure 1: Flow cytometry analysis of control and etoposide-treated MDA-MB-231 cells, 48 hrs post-treatment, showing the percentage of cells in each quadrant based on Annexin V/PI staining.

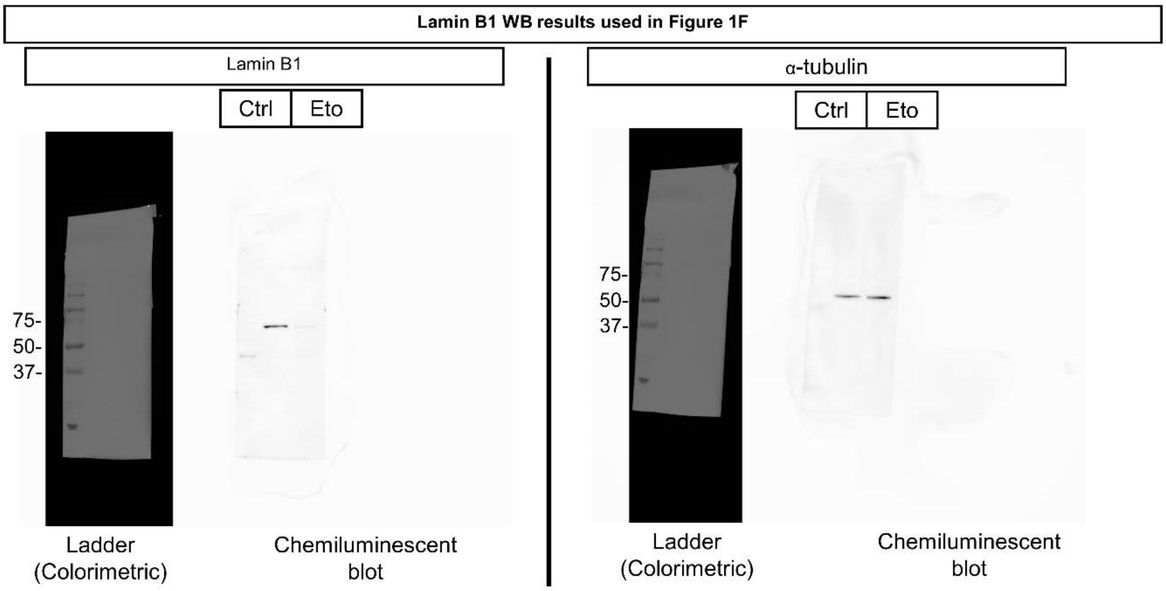

Suppl. Figure 2: Full Western blot showing Lamin B1 (67 kDa) expression in control and etoposide-treated MDA-MB-231 cells, with α-tubulin (55 kDa) included as a loading control.

Expression of fibronectin in decellularized proliferating and senescent cells on day 6 post-treatment

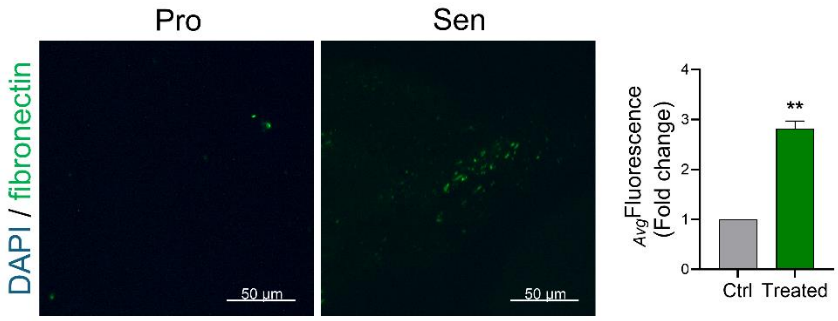

Suppl. Figure 3: Representative images and quantification of fibronectin (green) on decellularized coverslips. DAPI was used for counterstaining to detect any remaining cells. The graph represents the average of the total fluorescence intensity per area.

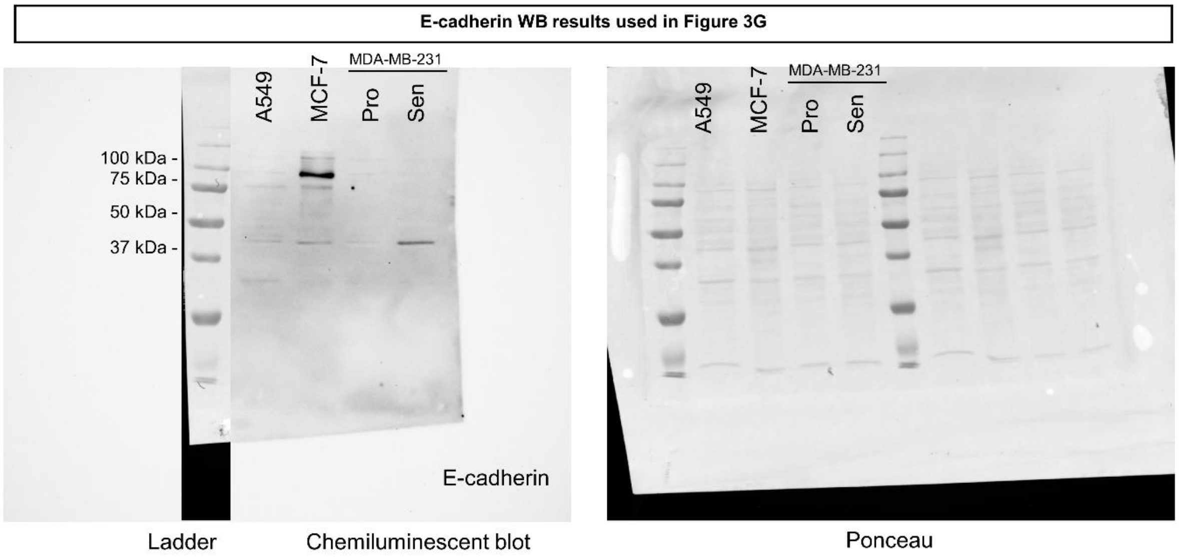

Suppl. Figure 4: Full Western blot showing E-cadherin antibody validation and expression profile in proliferating (Pro) and senescent (Sen) MDA-MB-231 cells, with Ponceau staining included as a loading control. The antibody was tested and validated using total protein lysates from A549 and MCF-7 cells to highlight cell line–specific differences in E-cadherin migration. While full-length E-cadherin (~120 kDa) was readily detected in MCF-7 cells, MDA-MB-231 cells displayed a band at approximately 43 kDa, consistent with a cleaved fragment of E-cadherin.

E-cadherin WB results used in Figure 3I

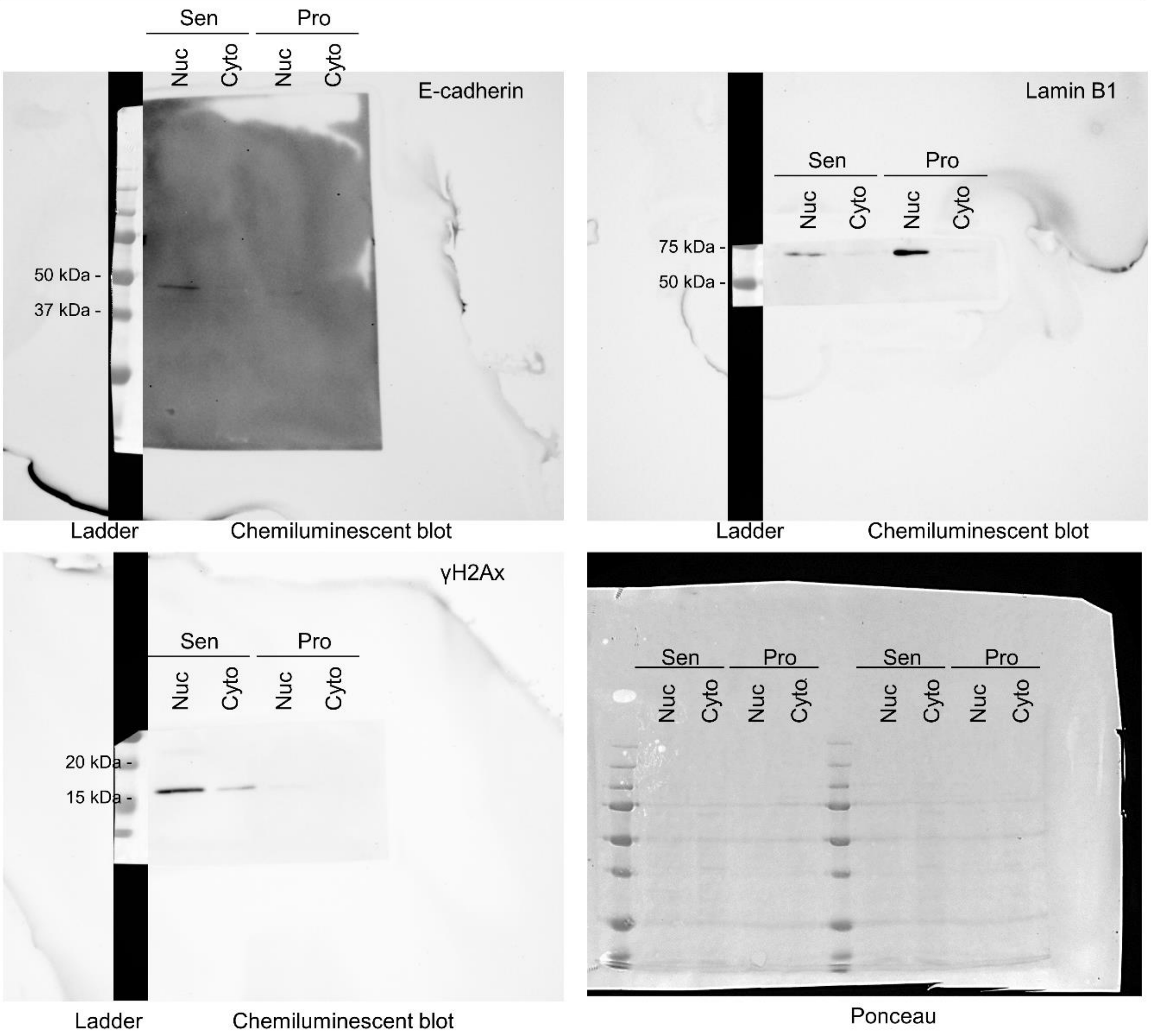

Suppl. Figure 5: Full Western blot showing E-cadherin expression in nuclear fractions of senescent (Sen) MDA-MB-231 cells, but not in their proliferating (Pro) counterparts. γH2AX and Lamin B1 blots were used as controls for subcellular fractionation, while Ponceau staining was included as a loading control.

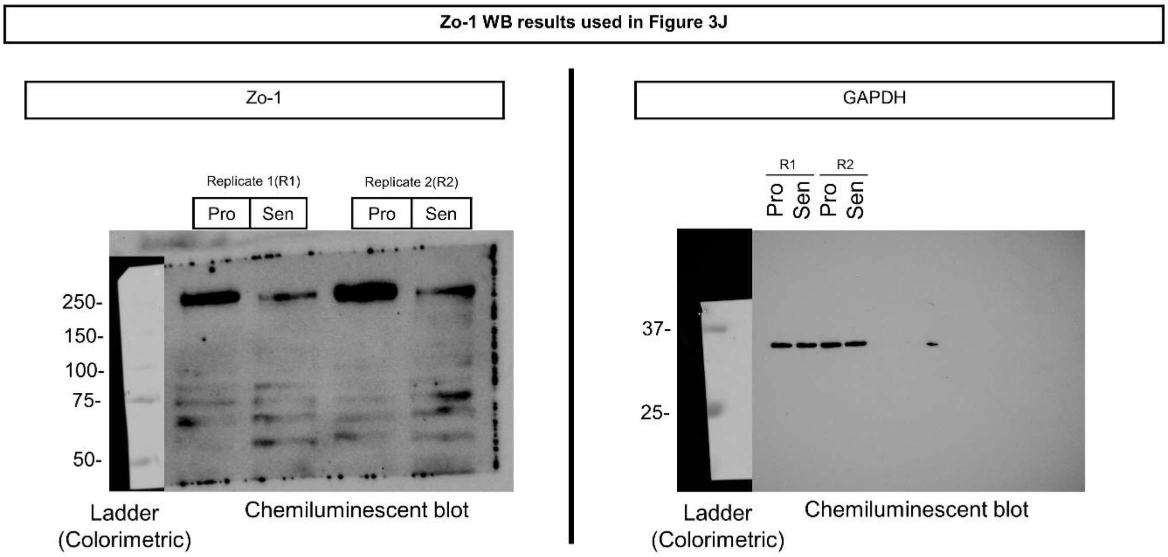

Suppl. Figure 6: Full Western blot showing Zo-1 (250 kDa) expression in proliferating (Pro) and senescent (Sen) MDA-MB-231 cells, with GAPDH (36 kDa) included as a loading control.

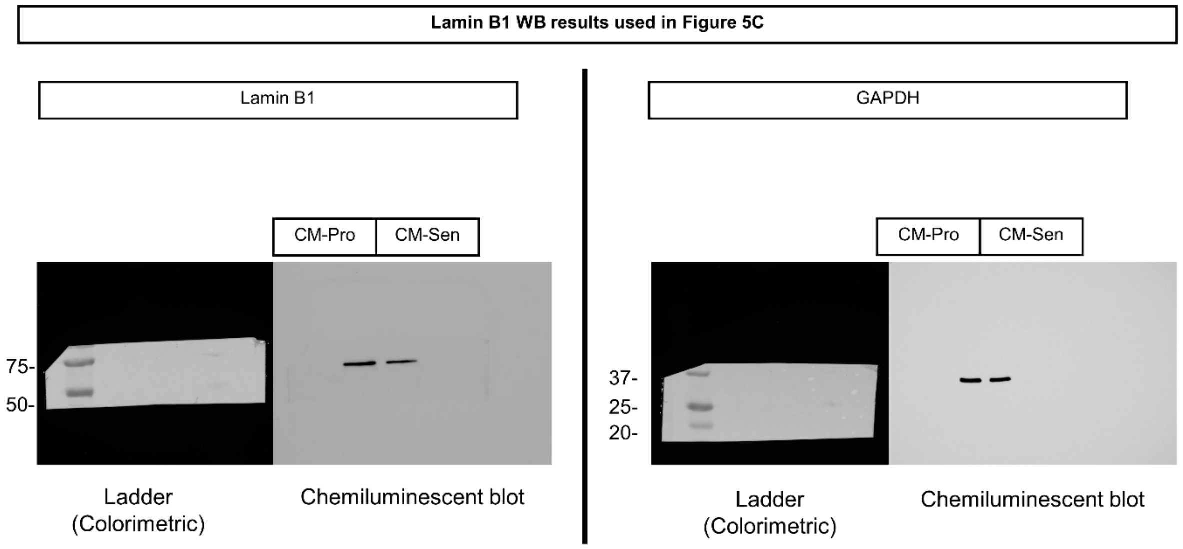

Suppl. Figure 7: Full Western blot showing Lamin B1 (67 kDa) expression MDA-MB-231 cells treated with CM from proliferating and senescent cells, with GAPDH (36 kDa) included as a loading control.
